# Role of the Orphan Nuclear Receptor NR4A Family in T-Cell Biology

**DOI:** 10.3389/fendo.2020.624122

**Published:** 2021-02-01

**Authors:** Livia Odagiu, Julia May, Salix Boulet, Troy A. Baldwin, Nathalie Labrecque

**Affiliations:** ^1^ Laboratory of Immunology, Maisonneuve-Rosemont Hospital Research Center, Montreal, QC, Canada; ^2^ Département de Microbiologie, Infectiologie et Immunologie, Université de Montréal, Montreal, QC, Canada; ^3^ Department of Medical Microbiology and Immunology, University of Alberta, Edmonton, AB, Canada; ^4^ Département de Médecine, Université de Montréal, Montreal, QC, Canada

**Keywords:** NR4A nuclear receptor, CD4 T cell, CD8 T cell, thymus, immune response, Nur77, Nurr1, Nor1

## Abstract

The nuclear orphan receptors NR4A1, NR4A2, and NR4A3 are immediate early genes that are induced by various signals. They act as transcription factors and their activity is not regulated by ligand binding and are thus regulated *via* their expression levels. Their expression is transiently induced in T cells by triggering of the T cell receptor following antigen recognition during both thymic differentiation and peripheral T cell responses. In this review, we will discuss how NR4A family members impact different aspects of the life of a T cell from thymic differentiation to peripheral response against infections and cancer.

## Introduction to T-Cell Biology

T cells are central players of the adaptive immune response. They recognize, *via* their T cell receptor (TCR), a peptide fragment of antigen (Ag) in association with class I or II molecules of the major histocompatibility complex (MHC). The generation of a repertoire of T cells endowed with the ability to recognize almost all the possible foreign Ags is possible due to TCR gene rearrangement, a process where random juxtaposition of TCR gene segments occurs to create TCR sequence diversity. This requires that developing thymocytes undergo an education process during their differentiation. Therefore, only thymocytes expressing a useful TCR (eventually able to recognize a foreign Ag in association with self-MHC molecules) will survive (positive selection) during differentiation while those expressing an auto-reactive TCR will be physically or functionally eliminated from the repertoire (negative selection). This stringent selection process ensures that only useful (MHC restricted) and self-tolerant T cells will colonize lymphoid organs as naïve T cells. The molecular events controlling thymic T cell differentiation and selection are still not fully understood. The first part of this review will highlight how deciphering the role of NR4A family members has helped to better understand the T cell differentiation events taking place in the thymus.

The detection and engulfment of pathogens by dendritic cells (DCs) within the tissue will induce their maturation and the presentation of peptide fragments from the pathogens within MHC class I or class II molecules expressed at their surface. These DCs will then migrate to the draining lymphoid organs where they will activate Ag-specific T cells. For efficient activation and differentiation into effector T cells able to control the infection, naïve T cells require three signals: TCR stimulation, co-stimulatory signals provided by mature DCs *via* CD28-CD80/CD86 interactions, and an inflammatory milieu (cytokines produced by DCs or the environment). This will lead to massive expansion of T cells to increase their numbers. Concomitant with T cell proliferation, differentiation will occur leading to the acquisition of effector functions crucial for the elimination of the infectious agent. After clearance of infection, most Ag-specific T cells will die by apoptosis while a few will survive and differentiate into memory T cells that will confer long-lived protection against reinfection. A different picture emerges in the context of chronic infection or cancer where the persistence of Ags and inflammation lead to a state of T cell exhaustion. In the second part of this review, we will present how the study of the role of the orphan nuclear receptor NR4A family members has provided a better understanding of the molecular events controlling peripheral T cell responses to infection and cancer.

## Overview of NR4A Orphan Nuclear Receptors

The NR4A family of orphan nuclear receptors is composed of NR4A1 (Nur77), NR4A2 (Nurr1), and NR4A3 (Nor-1). They work as transcription factors in a ligand-independent manner. Like other nuclear receptors, they are composed of a central two-zinc DNA-binding domain, a N-terminal transactivation domain, and a C-terminal ligand-binding domain (LBD). The LBD lacks a classical hydrophobic binding pocket, explaining ligand-independent action. They recognize the NBRE motif (AAAAGGTCA) on DNA as monomers and they can bind as homodimers to the palindromic DNA binding motif, NurRE (TGATATTTX_6_AAATGCCCA) (1, 2). Their functions are mostly controlled by the rapid and transient induction of their expression by a variety of extracellular signals, and thus are considered as immediate-early genes. The NR4As are involved in various cellular functions including apoptosis, survival, proliferation, angiogenesis, inflammation, DNA repair, and fatty acid metabolism (3, 4).

## NR4As and Thymic T Cell Development

### Overview of T Cell Development

The thymus is organized into two distinct regions; an outer cortical area and an inner medullary area that are composed of different cell populations. During T cell selection in the thymus, thymocyte fate is largely determined by the affinity of the TCR for self-peptide presented in the context of MHC molecules (spMHC). In the cortex, the generation of the αβ-TCR through random somatic recombination processes leads to the formation of a large pool of CD4^+^CD8^+^ double-positive (DP) thymocytes that express a highly diverse TCR repertoire. DP thymocytes that receive low affinity TCR signals undergo positive selection and lineage commitment, and traffic to the thymic medulla where maturation to the CD4^+^ or CD8^+^ single positive (SP) lineage is completed (5, 6). Upon receipt of a high affinity TCR signal, self-reactive thymocytes undergo negative selection which includes apoptosis induction (clonal deletion) or functional inactivation (anergy). Alternatively, thymocytes that receive strong TCR signals can undergo agonist selection and be diverted into nonconventional lineages such as T regulatory cells (Treg), invariant natural killer T cells (iNKT), or CD8αα^+^ intestinal intraepithelial lymphocytes (IEL) (7). In the thymic cortex, developing thymocytes encounter self-peptides derived from ubiquitously expressed proteins (6). It is necessary to remove T cells expressing autoreactive TCR directed against all self-proteins, including the ones whose expression is tissue-restricted. Therefore, in the thymic medulla and at the single-positive (SP) stage of thymocyte differentiation, thymocytes will encounter a different repertoire of self-peptides which includes those derived from proteins expressed in a tissue-restricted manner and driven by the promiscuous transcriptional activities of Aire and Fezf2 (8, 9).

**Figure 1:**
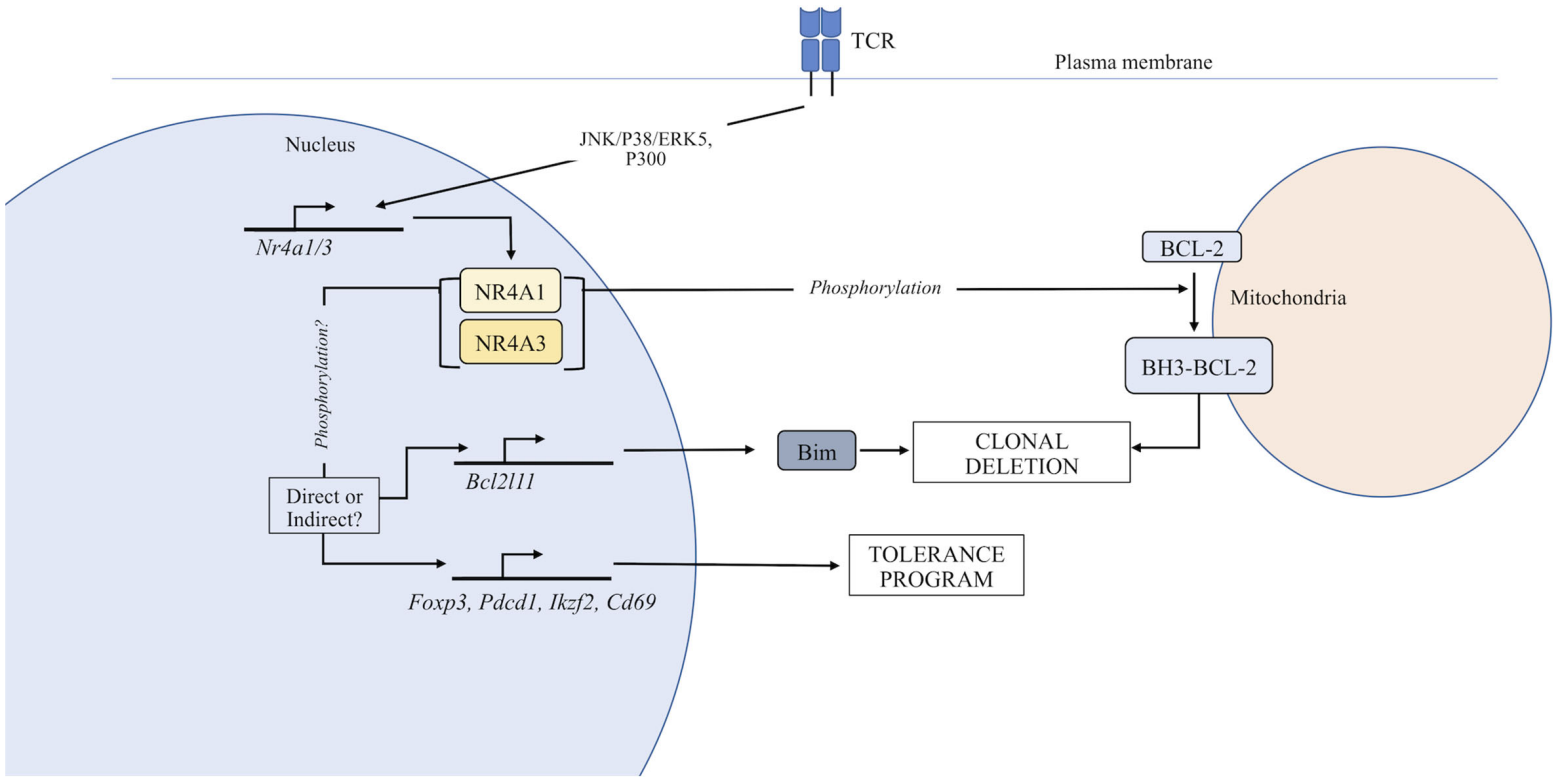
Function of the NR4A family in thymocyte development. TCR stimulation induces expression of the NR4A family. In the nucleus, NR4As can regulate the expression of genes that control T cell tolerance. Additionally, NR4A family members can be exported from the nucleus where they directly regulate apoptosis through an interaction with Bcl-2.

TCR signals received by nascent thymocytes lead to transcriptional changes that regulate positive and negative selection (10). Among the set of genes consistently associated with clonal deletion is NR4A1 (10, 11). The NR4A family has long been investigated for their putative role in thymocyte selection. NR4A1 and NR4A3, but not NR4A2, are expressed in thymocytes undergoing selection (12), but NR4A1 is the most extensively studied of the three NR4As and will receive the most attention in this section of the review. An initial connection between the NR4A family of proteins and thymocyte selection developed when NR4A1 induction was demonstrated in apoptotic immature thymocytes and T cell hybridomas (13, 14). Subsequent studies on the role of the NR4A family in thymocyte development utilized many different approaches and model systems which will be explored in the following sections. Additionally, while there is clear redundancy within the family, it is becoming apparent that phenotypic and functional changes in thymocyte development can be observed when the expression of individual family members is manipulated. This is further emphasized by emerging evidence suggesting that the induction of individual NR4A family members is differentially regulated downstream of TCR signaling (15). Added complexity stems from the fact that the function of NR4A family members during thymocyte development has been reported to be dependent on transactivation (16–19) or extra-nuclear activities (20–23) (**Figure 1**). Finally, this section will focus on the role of the NR4A family in αβ-thymocyte selection, since to our knowledge the NR4As have not been reported to regulate the development of any other thymic lineages.

### Cortical Negative Selection to Ubiquitously Expressed Self-Antigen

The thymic cortex is the compartment in which nascent thymocytes that express an αβ-TCR first encounter spMHC, and where both positive and negative selection are known to occur (6, 7). DP thymocytes are selected based on the affinity of the TCR for “ubiquitous” self-Ag (UbsA) presented in the context of MHC on the surface of cortical thymic epithelial cells (cTEC) and DCs (6). NR4A1, NR4A3 (24), and NR4A2 (25) share variable sequence homology in the N-terminal transactivation domain and C-terminal “ligand binding domain,” but close similarity in their DNA-binding domains. Original studies implicating the NR4As in thymocyte clonal deletion demonstrated that a NR4A1 dominant negative mutant that lacked the transactivation domain or antisense NR4A1 RNA inhibited TCR-induced cell death in T cell hybridomas (13, 14, 26, 27). However, it was proposed early on that T cell hybridomas more closely model the responses of mature T cells than developing thymocytes (28, 29). *In vivo* models of negative selection to ubiquitous antigen were subsequently employed. Dominant negative NR4A1 expressed in HY TCR transgenic mice, wherein thymocytes recognize the ubiquitous male-specific HY antigen in the context of H-2D^b^ (30, 31), resulted in a partial rescue of DP and CD8SP populations from clonal deletion; however, tolerance to HY male antigen was maintained. Similar results were observed for models that involved injection of exogenous antigen to induce thymic clonal deletion such as in F5 TCR transgenic mice (specific for influenza nucleocapsid peptide) (27, 30, 32). More contemporary studies investigating the transcriptional regulation of NR4As provided additional support for the NR4A family in regulating negative selection and tolerance. In pre-selection DP thymocytes, histone deacetylase 7 (HDAC7) in complex with MEF2D represses NR4A1 and NR4A3 expression (33, 34). Following TCR signaling, HDAC7 is phosphorylated and exported from the nucleus, discontinuing its repressive activity (33–36). Transgenic mice harboring an HDAC7 mutant putatively incapable of being phosphorylated downstream of the TCR and exported from the nucleus (S155/318/448A) showed impaired induction of NR4A1 and NR4A3, impaired negative selection, and lethal autoimmunity (37). While this may indicate the importance of NR4A1/NR4A3 in clonal deletion, the cause of autoimmunity in this model is unclear, especially since these HDAC7 mutants demonstrated a generalized suppressive impact on the negative selection transcriptional program and impaired generation of Tregs (37).

As a result of the similarity among the NR4As in their DNA-binding domains, dominant negative mutants of individual NR4As have the potential to inhibit the transactivation activities of other NR4A family members (12). Therefore, studies employing the NR4A1 dominant negative mutant have provided support for the hypothesis that NR4A receptors drive thymocyte clonal deletion *via* their transcriptional activity (38–40). In harmony with this assertion, overexpression of full-length NR4A1 or NR4A3 (but not NR4A2) induced thymocyte apoptosis *in vivo*, while that of the NR4A1 dominant negative did not (12, 27, 30, 41) but this assumes that the dominant negative NR4A1 mutant only impairs the transcriptional activity of NR4A1. Transcriptional targets of NR4A1 thought to potentially mediate its pro-apoptotic effect were FasL, TRAIL, and Nur77 downstream gene 1 and 2 (NDG1/2) (40). While none of these targets were shown to be required for the thymocyte apoptosis induced by a full-length NR4A1 transgene, we caution that thymic phenotypes in NR4A transgenic mice may not accurately represent endogenous NR4A function during thymocyte selection since NR4A transgenes were active during the DN stage (e.g., Lck proximal promoter), not the DP stage where negative selection first occurs (15, 39, 42, 43).

Conversely, NR4A1/3 have been proposed to induce apoptosis by their nuclear export and conversion of anti-apoptotic Bcl-2 to a pro-apoptotic form *via* exposure of its Bcl-2 homology domain 3 (BH3) at the mitochondria in a variety of cell types including thymocytes (20–23). Bcl-2/BH3 conversion has been shown to occur *in vivo* in the HY and F5 TCR transgenic UbsA selection models specifically within DP, but not SP thymocytes, implying this NR4A activity is unique to UbsA-mediated negative selection (21). A study that used a Bcl-2 transgene wherein the key amino acid residues critical for the pro-apoptotic function of the Bcl-2 BH3 domain were mutated demonstrated that expression of the BH3-mutant Bcl-2 enhanced rescue of high affinity and specific Vβ TCR-expressing clones experiencing negative selection to endogenous superantigen compared to wildtype Bcl-2. This indicated that pro-apoptotic conversion of Bcl-2 may be a mode of influence for NR4A1/NR4A3 on clonal deletion. However, subsequent work using the HY^cd4^ TCR transgenic model, which is specifically designed for physiological timing of expression of the αβ-TCR in contrast to traditional TCR transgenics (44), failed to observe exposure of the Bcl-2 BH3 domain during negative selection (18).

As a better approach to study the function of NR4A1 in thymocyte development, a NR4A1 knock-out (KO) mouse was generated. NR4A1 deficiency did not impair clonal deletion in the HY TCR and AND TCR transgenic models of negative selection to UbsA (28). The dispensability of NR4A1 for UbsA-mediated clonal deletion was further reinforced by a study using the physiological HY^cd4^ transgenic TCR model (18). Due to putative redundancy in the transcriptional activities of the NR4As mentioned above, the lack of phenotype in NR4A1-deficient mice has long been thought to be due to compensation by the remaining intact NR4A family member NR4A3 (12), and has received support from the study of the NR4A family in Treg development (45) (see below). However, even though NR4A1 deficiency did not impair clonal deletion, it altered the expression of proteins induced during negative selection to UbsA such as PD-1, Helios, and CD69 demonstrating a NR4A1 transcriptional footprint on UbsA-mediated negative selection (18, 40, 46).

Against complete functional redundancy of NR4A1/3 is the differential regulation of NR4A family member expression. NR4A1 is induced in thymocytes receiving both positive and negative selection signals, though to a greater degree for the latter (10, 42), while NR4A3 expression appears to be induced only by high affinity signals (15). This supports the notion that the different NR4A family members can differentially contribute to thymocyte selection events. NFAT has been proposed necessary for the induction of NR4A3 (but not NR4A1 expression) in CD4^+^ and CD8^+^ peripheral T cells, especially in the context of exhaustion (15, 47, 48). Meanwhile, ERK signaling is required for optimal NR4A1 and NR4A3 induction in peripheral T cells (based on chemical inhibitor experiments) (15). Most recently, a specific pathway has been proposed for NR4A1 regulation in thymocytes, wherein ASK1-JNK/P38 MAP kinases promote the induction of NR4A1, while Fas apoptotic inhibitory molecule (FAIM) and Akt inhibit this cascade (49). Some additional proposed positive regulators of NR4A1 and NR4A3 induction are P300, MEF2D, and ERK5 (2, 50).

### Negative Selection to Tissue-Restricted Self-Antigen Expressed in the Medulla

Following positive selection in the thymic cortex, developing thymocytes traffic to the medulla *via* upregulation of the CC-chemokine receptor 7 (CCR7) coordinated with maturation to the SP stage (5). Within this compartment, negative selection is induced by a new “tissue-restricted” self-antigen **(TRsA)** repertoire regulated by the transcription factors AIRE and Fezf2 and mediated by a distinct complement of antigen presenting cells (6, 9, 51). Investigation of *in vivo* negative selection to TRsA revealed that NR4A1 contributes to clonal deletion in a manner not fully compensated for by NR4A3. Using the MHC II-restricted OT-II RIP-mOVA model, Fassett et al. showed that deficiency in NR4A1 impaired clonal deletion of CD4SP transgenic thymocytes (46). In this model, the OT-II transgenic TCR recognizes a peptide derived from the chicken protein, ovalbumin (OVA) (52) and OVA is expressed in both pancreatic beta cells and thymic medulla owing to its control by the rat insulin promoter (53). Additionally, Fassett et al. showed that combined deficiency in NR4A1 and Bim, a key inducer of thymocyte apoptosis (54), did not further impair clonal deletion compared to NR4A1-deficiency alone. From a mechanistic perspective, they found a reduction in mRNA levels of *Bcl2l11* (coding for Bim) in the absence of NR4A1, suggesting that NR4A1-mediated induction of Bim was at least one way NR4A1 regulated clonal deletion (46). This contrasts with a study using the OT-I TCR RIP-mOVA transgenic model (55), in which Bim does not require NR4A1 for its transcriptional induction (19). In this model, deficiency in NR4A1 only modestly impaired clonal deletion as evidenced by a small increase in mature OT-I thymocytes. Single deficiency in either NR4A1 or Bim did not result in broken tolerance, however, combined deficiency resulted in broken self-tolerance signified by the development of diabetes. Since the number of mature OT-I thymocytes and T cells was similar in Bim-deficient and NR4A1/Bim doubly deficient situations, the break in tolerance suggests NR4A1 regulates tolerance through altering the functional capabilities of the thymocytes or T cells (19). A role for NR4A3 and redundancy between NR4A1 and NR4A3 has not been investigated in a medullary antigen-specific model system.

How do NR4A family members regulate medullary negative selection? In addition to regulating the expression of Bim as seen in the OT-II Rip-mOVA model, nuclear export of NR4A1 following “death signals” (e.g., etoposide, calcium ionophore, phorbol ester, TCR signal) has been observed and appears to occur following the phosphorylation of a serine residue (S354 in mouse) in the DNA binding domain (20, 23, 38, 56, 57). The localization of highly phosphorylated NR4A1 to the cytosolic fraction has also specifically been associated with SP, but not DP thymocytes stimulated *in vitro* with TCR- and CD28-specific plate-bound antibodies (38). While S354 of NR4A1 has been proposed as a target residue for Akt and the ERK1/2-RSK pathway (23, 57, 58), there is debate as to whether phosphorylation at this site mediates nuclear export or retention (23, 38, 59); however, it appears to vary across model systems and cell types (23, 59–61). In thymocytes, protein kinase C (not AKT, JNK, ERK1/2 or p38) is thought to phosphorylate both NR4A1 and NR4A3, leading to mitochondrial translocation (59), yet both MAPK and PI3K inhibitors have been shown to inhibit this phenomenon specifically in SP thymocytes in a separate study (38). Nuclear export would thus be consistent with a role for NR4A1 in directly mediating TRsA-induced clonal deletion in the OT-II model (46), if NR4As indeed drive clonal deletion by translocation to mitochondria. However, deficiency in Bim alone impaired clonal deletion in this model (62), suggesting that in this case NR4A1 is not sufficient to drive clonal deletion independently of Bim, lending further support for NR4A transcriptional activity as the major modulator of thymocyte fate. In both thymocytes and T cell hybridomas, the MEK5-ERK5 pathway has been proposed to regulate thymocyte apoptosis by both inducing NR4A1 expression downstream of the TCR signal (50, 63), and phosphorylating NR4A1 leading to enhanced NR4A1 transcriptional activity (64). In further support of the concept of diverging functions of the NR4As across the stages of thymocyte development (and in UbsA- and TRsA-mediated negative selection), Akt is thought more active in DP than SP thymocytes (38, 65), and thought to direct the ubiquitination and degradation of NR4A1 in both T cell hybridomas and thymocytes (66). However, the relationship between NR4A post-translational modification and function, whether it be mitochondrial translocation or transactivation (or perhaps a combination of both), requires further investigation using *in vivo* model systems paying close attention to discrete T cell subsets. Overall, no consistent dependence on induced pro-apoptotic genes has been observed across studies. As a result, an emerging view is that the NR4As may influence selection outcomes by transcriptionally modulating the T cell tolerance program (18, 46, 67–70). Supporting evidence includes an increased susceptibility in NR4A1 deficient mice for experimental autoimmune encephalomyelitis (EAE; 2D2 transgenic TCR model), allergic contact dermatitis, collagen-induced arthritis (69), and diabetes (19). However, it is unclear whether this increased autoimmunity is due to changes in thymocyte development or peripheral T cell function. Studies employing conditional NR4A1 knock-out models will be necessary to resolve this question.

### Positive Selection/Lineage Commitment

Despite induction of NR4A1 during positive selection (10, 42), neither it nor the other NR4As have been shown to be required for this process. As was discussed in the preceding section, there are differences observed between MHC I- and MHC II-restricted models of negative selection, which suggest the NR4As may play diverging roles in the selection of CD8^+^ and CD4^+^ T cell lineages. This view is reinforced by the observation that both CD4SP thymocytes and mature CD4^+^ T cells from a polyclonal repertoire express elevated basal levels of a Nur77-GFP reporter compared to CD8^+^ lineages, perhaps owing to the proposed enhanced signal delivered by the CD4 coreceptor compared to that of CD8 (42, 71, 72). In polyclonal thymocytes and peripheral T cells, NR4A1 has been shown to negatively regulate the transcriptional activation of *Runx3*, a critical operator of CD8^+^ T cell lineage commitment, which may alter the relative stability of selection into the CD8^+^/CD4^+^ lineages (43, 73). However, RNA-sequencing analysis of NR4A triple-KO CAR-transduced CD8^+^ T cells revealed no effect on *Runx3* expression, perhaps suggesting diverging influences by individual NR4As (67) or differences in thymocytes versus mature T cells. Nevertheless, deficiency in both NR4A1 and Bim led to enhanced efficiency of positive selection in female mice bearing the MHC I-restricted HY^cd4^ transgene (18) and NR4A1 deficiency alone resulted in increased positive selection in the OT-II TCR transgenic model (46). However, NR4A1 deficiency did not enhance positive selection in the OT-I transgenic model (19). Overall, the contribution of NR4A1 to positive selection requires further examination and remains unclear, though its influence may vary with selection circumstances including lineage commitment and the intrinsic self-reactivity of transgenic TCR models investigated.

### Alternate Thymocyte Fates

It is clear the NR4As do not always function as proapoptotic mediators. Indeed, the NR4As have been implicated in non-apoptotic processes following strong TCR signaling such as the development of non-conventional CD4^+^ fates (Treg and anergic CD4^+^ T cell) (19, 45, 46, 74, 75). Treg are a distinct lineage of T cells that are selected in the thymus and are critical for tolerance. Lineage specification and function of Treg is controlled by expression of the transcription factor Foxp3. Each NR4A member has been implicated in variably promoting the activation of *Foxp3* transcription and other genes associated with the Treg signature (e.g., *Ikzf4* and *Il2ra*) (17, 19, 46, 75). As such, triple NR4A deficiency resulted in the loss of Tregs in thymic and peripheral compartments, systemic multiorgan autoimmunity, and a skewing of the mature CD4^+^ T cell repertoire toward an activated phenotype (CD44^hi^ CD62L^lo^) (45). Combined NR4A1 and NR4A3 deficiency nearly recapitulated the phenotype of the triple knock out, thus NR4A2 may not play as key a role in Treg development and homeostasis. It should be mentioned, however, that mice lacking all three NR4As displayed generalized thymic abnormalities, which may connote defects independent of the Treg compartment or could result from excessive inflammation. A more recent study has provided evidence for a positive feedback loop between the NR4A family members and Foxp3 which involves reciprocal promoter binding and transactivation, and functions to reinforce Treg development from the CD25^+^ Foxp3^−^ CD4^+^ precursor stage (45, 74, 75). Despite its putative ability to transactivate *Foxp3*, deficiency in NR4A1 has been shown to enhance selection efficiency of the natural Treg (nTreg) lineage, suggesting that NR4A contributions to agonist selection extend beyond the direct transactivation of lineage-associated genes (19, 46). In both a polyclonal and OT-II transgenic context, deficiency in NR4A1 alone resulted in enhanced selection of CD4SP thymocytes into the Foxp3^+^ Treg lineage and the early CD25^+^ Foxp3^−^ CD4^+^ Treg precursor subset (18, 46) in a cell-intrinsic manner, the former of which displayed a normal transcriptional footprint and suppressive activity (46). These findings are intriguing as they suggest that in the absence of NR4A1 other NR4A family members are sufficient to drive Foxp3 expression, and that in addition to promoting Foxp3 expression, NR4A inhibits selection into the Treg lineage. Future studies are required to determine how NR4A1 negatively regulates Treg selection.

An additional outcome of high affinity TCR signaling is the generation of anergic phenotype FR4^hi^ CD73^hi^ CD4^+^ T cells, which are also thought a precursor to Foxp3^+^ Tregs (76–78), and which demonstrates enhanced thymic development in a NR4A1-deficient context (18). While this may be attributable to the cell-extrinsic generation of anergic phenotype CD4^+^ T cells by Foxp3^+^ Tregs, these anergic phenotype CD4^+^ T cells may also feed into the Foxp3^+^ Treg population as precursors when NR4A1 is deficient (18, 78). How NR4A1 impacts the relationship between Treg and anergic phenotype CD4^+^ T cells requires further investigation. Finally, NR4A1 transgenic overexpression has most recently been associated with driving developing iNKT cell apoptosis and an “exhausted” phenotype in a cell-intrinsic and extrinsic manner (79). Continued study into the role of the NR4As in iNKT development and function may prove critical to understanding iNKT-driven autoimmune responses.

### Future Perspectives

Given the reported differences in NR4A family member function across different MHC-restricted models and systems modeling UbA- and TRA-mediated negative selection, it appears probable that the NR4As perform multiple specific roles—both nuclear and extranuclear—within discrete lineages and stages of T cell development. Differences observed between MHC I- and MHC II-restricted TCR models could be due to the differential selection contexts determined in part by the type of antigen presenting cell (APC). This would include antigen processing and presentation efficiency and associated co-stimulatory molecules (6). In future studies, the importance of heeding differences in model systems is thus apparent. In this regard, the non-physiological timing of typical TCR transgenes may introduce additional difficulty in interpreting the roles of the NR4As (44, 79, 80). In addition, the classically utilized NR4A1 “knock-out” results in the translation of the N-terminal 117 amino acids of NR4A1, which is not present with Cre-Lox removal of the NR4A1 translational start codon. This truncated NR4A1 is not inert, but is in fact associated with liver immune infiltration, loss of splenic architecture, and altered hematopoietic stem cell homeostasis (81). The N-terminal region of NR4A1 has been shown to inhibit the MDM-2 induced degradation of HIF-1α, which regulates HSC mobilization (82, 83). Whether this feature of the germline NR4A1 KO influences thymocyte development is unclear, but suggests additional studies using the conditional NR4A1 KO be considered.

## NR4As and Peripheral CD8^+^ T Cell Responses

It was rapidly realized that NR4A expression was not only induced in thymocytes following TCR stimulation but also in peripheral mature T cells. Their possible role in T cell response was first suggested by the identification of a correlation between their expression and the ability of T cells to differentiate into memory T cells (84, 85). Recent studies have revealed an important role for NR4As during acute and chronic CD8^+^ T cell responses (**Table 1**).

**Table 1:** Role of NR4A family members in CD8 T cells.

Expression	*Nr4a1*	*Nr4a2*	*Nr4a3*	References
Rapid and transient induction by *in vitro* TCR stimulation	✓	✓	✓	(42, 85–90)
Rapid and transient induction during *in vivo* acute response	✓	✓	✓	(42, 87, 89, 91)
Constitutive expression by resident memory T cells	✓	✓	✓	(92–95)
High expression by exhausted T cells	✓	✓	✓	(47, 48, 67, 96–99)
				
**Function**	**NR4A1**	**NR4A2**	**NR4A3**	**References**
T cell proliferation	ê	N.D.	No effect	(68, 88, 89)
Cytokine production	ê	N.D.	ê	(67, 68, 88, 89)
SLEC differentiation	ê or no effect	N.D.	é	(68, 88, 89)
MPEC differentiation	é or no effect	N.D.	ê	(66, 88, 89)
Central memory T cell generation	No effect	N.D.	ê	(89, 100)
Resident memory T cell generation	é	é	é	(94, 95, 100)
T cell exhaustion	é	é	é	(48, 67, 88, 89)
				
**Molecular mechanism**	**NR4A1**	**NR4A2**	**NR4A3**	**References**
Competition for bZIP transcription binding activity	✓	N.D.	✓	(67, 89)

CD8^+^ T cells are potent cells of the adaptive immune system able to eradicate intracellular infections, control chronic infections, and eliminate tumors. The success of a primary immune response to acute infection requires proper control of cell fate to generate a large number of short-lived effector cells (SLECs; CD127^lo^KLRG1^hi^) that will control the pathogen and memory precursor effector cells (MPECs; CD127^hi^KLRG1^lo^) that differentiate into long-lived memory CD8^+^ T cells to confer long-term protection. In chronic infection or cancer, where antigen and inflammation persist, T cell exhaustion is associated with an expression of inhibitory receptors (PD1, Tim3, 2B4, Lag3, etc.) and a progressive loss of T cell functions (101). During chronic response, exhausted CD8^+^ T cells can be divided into stem-like, transitory, and terminally differentiated subsets (102–104). The restoration of T cell functions following checkpoint blockade (e.g., anti-PD1 or anti-PD-L1) has been reported to act on the stem-like subsets.

### Expression of NR4As During Acute CD8^+^ T Cell Responses

Studies showing that *Nr4a* gene transcription was induced in thymocytes by TCR signaling raised the possibility that this could similarly occur in mature CD8^+^ T cells. Using the NR4A1-GFP reporter mouse model, Moran et al. have shown that only TCR signaling, not inflammatory signals, can rapidly induce *Nr4a1* transcription both *in vitro* and *in vivo* and that the level of GFP expression is proportional to the strength of TCR signaling (42). This reporter mouse model is now widely used to measure the *in vivo* timing and strength of TCR signaling in thymocytes and peripheral T cells as GFP^+^ cells are those that have recently received a TCR signal. The induction of the other NR4A family members during CD8^+^ T cell responses was first supported by the rapid and transient transcription of *Nr4a1*, *Nr4a2*, and *Nr4a3* during the immune response to *Listeria monocytogenes* infection (91) with a peak of expression at 12h post T cell activation and a return to baseline levels at 48h. This is consistent with induction by TCR signaling and suggests an early role for NR4As in CD8^+^ T cell response. Later on, it was shown using single cell RNA sequencing (scRNAseq) of *in vitro* antigen-stimulated CD8^+^ T cells that *Nr4a* genes transcription is weak in unstimulated CD8^+^ T cells, high at 1 and 3h post-TCR stimulation and already lower at 6h (86). Furthermore, the level of *Nr4a1* mRNA was proportional to the strength of TCR signaling (86). Finally, it was reported that NR4A1 expression is also an accurate and specific marker to identify human T cells that have recently been activated *via* their TCRs thus validating the use of *Nr4a1* induction as specific marker of recent TCR signaling (90).

The fact that *Nr4a3* is also induced following TCR stimulation has led to the development of *Nr4a3-*Tocky reporter mouse. Instead of GFP, Tocky reporter protein possesses time-dependent decay fluorescence shifting its emission from blue to red. This property allows for the observation of transient versus persistent TCR activation both *in vitro* and *in vivo* (87).

Although all these studies identified NR4As as early immediate genes induced by TCR signaling, the identification of the role of this induction in peripheral immune responses has only been recently uncovered.

### Contribution of NR4A Family Members During Acute CD8^+^ T Cell Response

Transcriptomic studies suggesting that NR4As may have an important role in the early stages of the CD8^+^ T cell response (91) were later supported by the analysis of the dynamics of chromatin accessibility following T cell activation (48, 105). Indeed, the NBRE motif was enriched in chromatin regions that are highly accessible following acute CD8^+^ T cell stimulation (48), as early as 2h post *in vitro* TCR activation (105). This enrichment was maintained up to 24h post-activation but was less apparent in *in vivo* effector CD8^+^ T cells (at day 7 post-infection) and memory CD8^+^ T cells (105). Altogether, this dynamically regulated chromatin accessibility from naïve to recently activated cells suggested a role for NR4A transcription factors during early CD8^+^ T cell activation, which was then revealed by different groups.

In a first study, *Nr4a1* ^−/−^ mice showed better CD8^+^ T cell proliferation following *in vitro* anti-CD3 or antigen stimulation. Similar enhancement of CD8^+^ T cell proliferation was observed *in vivo* after adoptive transfer of *Nr4a1* ^−/−^ CD8^+^ T cells into wild-type recipient followed by antigen administration or into lymphopenic MHC class I-deficient hosts (68). Furthermore, *ex vivo* production of IFN-γ by CD8^+^ T cells is increased in absence of NR4A1 (68). Using full body knock-out *Nr4a1* ^−/−^ mice, the authors also showed increased Ag-specific CD8^+^ T cell expansion, SLEC generation, and granzyme B production following infection with *Listeria monocytogenes* (68). Unfortunately, cytokine production or the generation of CD8^+^ T cell memory were not evaluated in this setting. A more recent study using adoptive transfer of *Nr4a1* ^−/−^ TCR transgenic CD8^+^ T cells followed by acute LCMV infection showed that NR4A1 deficiency increased CD8^+^ T cell expansion and function but did not affect MPEC/SLEC differentiation, although T-bet expression, a transcription factor important for SLEC generation, was increased (88). The discrepancy on the effect of NR4A1 on MPEC/SLEC differentiation between the two studies might be due to the use of a full body knock-out *versus* T cell specific deletion (68, 88). We recently demonstrated that NR4A3 also influences CD8^+^ T cell differentiation during acute response to *Listeria* and LCMV infection and vaccination (89). NR4A3 ablation in CD8^+^ T cells did not affect Ag-specific T cell expansion but did influence MPEC/SLEC differentiation and cytokine production. Indeed, *Nr4a3* ^−/−^ CD8^+^ T cells differentiated less into SLECs and more into MPECs, As a consequence, more central memory CD8^+^ T cells were generated in the absence of NR4A3 (89). Similar to *Nr4a1* ^−/−^ CD8^+^ T cells, NR4A3 deletion enhanced cytokine production (IL-2, IFN-γ, and TNF-α) by Ag-specific CD8^+^ T cells. Therefore, NR4A1 and NR4A3 have a similar impact on cytokine production but not on MPEC/SLEC differentiation, suggesting that these have redundant and non-redundant functions. Further studies should reveal if NR4A2 induction contributes to CD8^+^ T cell response.

While *Nr4a* are immediate early response genes that are rapidly induced following TCR activation, they are also transcribed by resident memory CD8^+^ T cells (Trm), a subset of memory T cells that establish permanent residency at the site of infection. The transcription of *Nr4a* genes is part of the gene signature characterizing CD8^+^ Trm cells (92, 93, 95) and a study aiming at identifying transcriptional factors involved in CD8^+^ Trm cell differentiation identified NR4As among possible important regulators (95). Indeed, in a pooled shRNA screen, CD8^+^ T cells containing shRNAs against *Nr4a1*, *Nr4a2*, and *Nr4a3* were less present in the Trm pool (95).

Boddupalli et al. formally demonstrated the importance of NR4A1 in Trm cells. In the context of influenza infection, NR4A1 deficiency in CD8^+^ T cells decreased the number of Trm cells in the liver, Peyer patches, and intestinal epithelial lymphocytes (IELs) without any effect on lungs or bone marrow CD8^+^ Trm cells (100). Other subsets of memory CD8^+^ T cells, effector memory (Tem) and central memory (Tcm), which do not transcribe *Nr4a1*, were not affected by NR4A1 deficiency, demonstrating a specific requirement for *Nr4a1* in Trm biology (100). This is in opposition to the role NR4A3 plays in the development of memory T cells, as we have observed enhanced CD8^+^ Tcm cell generation in the absence of NR4A3 (89). Interestingly, recent studies evaluating the heterogeneity of Trm cells following LCMV infection by scRNA-Seq have revealed that *Nr4a1*, *Nr4a2*, and *Nr4a3* are particularly enriched in the highly functional CD28^+^ subset of CD8^+^ Trm cells and that knockdown of *Nr4a2* specifically decreased the proportion of these CD28^+^ Trm cells (94). Further studies should be done to determine which signals mediate the expression of NR4A family members in CD8^+^ Trm cells and how they play a role in Trm cell differentiation.

**Figure 2:**
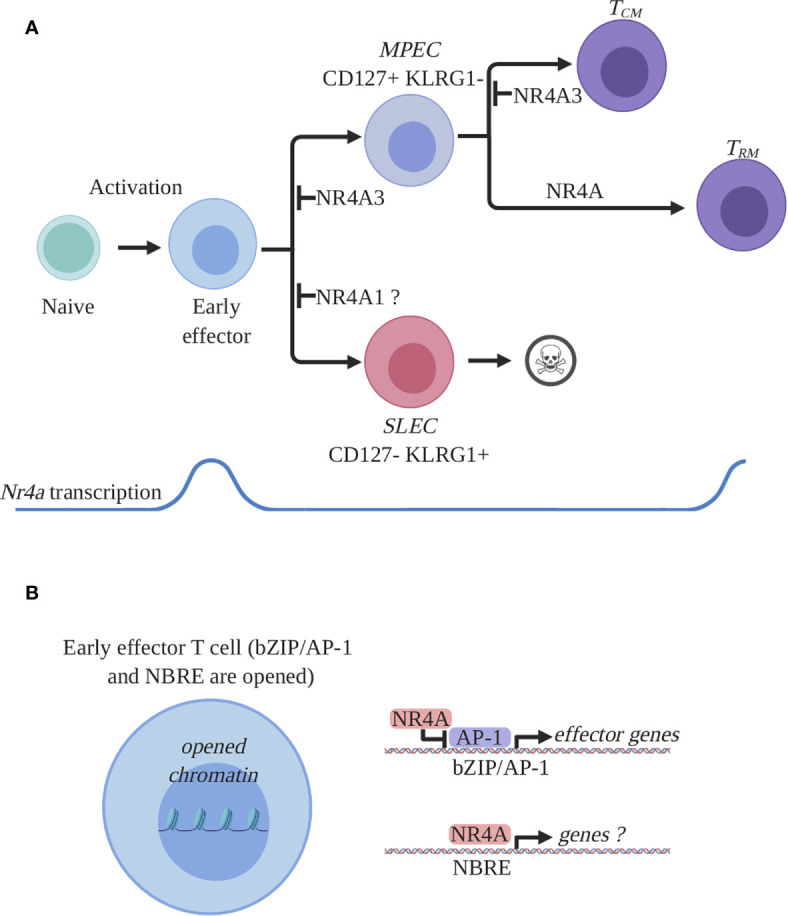
NR4A family members expression and function in CD8^+^ T cells during an acute immune response. **(A)** Expression and role of NR4A members in CD8^+^ T cell response. Antigen recognition by naïve CD8^+^ T cells will induce a transcriptional program responsible for activation, proliferation, and differentiation and proliferation. Among the activation-induced genes are all the *Nr4a* transcription factors which are rapidly and transiently induced at the early effector stage. Early effector CD8^+^ T cells will further differentiate into effectors endowed with the ability to control the infection. Two main subpopulations of effectors are generated: short lived effector cells (SLEC) and memory precursor effector cells (MPEC). SLECs will die by apoptosis following pathogen clearance while MPECs will survive and differentiate into memory T cells. At the effector stage, NR4A1 was shown to either inhibit or have no effect on SLEC differentiation while NR4A3 was shown to diminish MPEC differentiation. At the memory stage, the *Nr4a* transcription was shown to be enriched in a particular subset of memory CD8^+^ T cells, the resident memory CD8^+^ T cells (Trm). All the NR4A family members participate in the differentiation of CD8^+^ Trm cells while only NR4A3 was shown to influence central memory CD8 T cell (Tcm) differentiation. **(B)** Proposed molecular mechanism by which NR4A influences effector CD8^+^ T cell differentiation. CD8^+^ T cell activation will lead to the opening of the chromatin allowing for the transcriptional activity of different transcription factors involved in CD8^+^ T cell response. Among these transcription factors are bZIPs and NR4As which bind bZIP or NBRE DNA-binding motifs. Thus, bZIP TFs will occupy their recognition motifs on DNA and will drive the transcription of the effector- and differentiation-related genes. NR4A will influence CD8^+^ T cell transcriptional response by competing with bZIPs for DNA occupancy and by directly regulating genes containing NBRE motifs. The identity of the genes directly regulated by NR4A are still unknown. This figure was created with Biorender.com.

In summary, during an acute immune response, the expression of NR4As is rapidly induced and this contributes to CD8^+^ T cell response (**Figure 2A** and **Table 1**). At the effector stage both NR4A1 and NR4A3 reduce cytokine production, while only NR4A3 promotes SLEC differentiation. At the memory stage, *Nr4a* genes are selectively transcribed by CD8^+^ Trm cells with the three members possibly contributing to Trm cell differentiation while NR4A3 represses CD8^+^ Tcm cell generation. As not all NR4As were properly studied at each differentiation steps of CD8^+^ T cells, a full understanding of their respective role during acute CD8^+^ T cell response await further studies.

### Contribution of NR4A Family Members During Chronic CD8^+^ T Cell Response

One of the first studies that predicted involvement of NR4As in CD8^+^ T cell exhaustion was based on a model system called CA-RIT-NFAT where the NFAT protein was made constitutively active, but could not interact with the AP-1 transcription factor complex. This NFAT construct induces an exhausted/dysfunctional transcriptional program in CD8^+^ T cells (96). In this model, transcription of both *Nr4a2* and *Nr4a3* was upregulated. A subsequent study also pointed toward a role for NR4As in CD8^+^ T cell exhaustion during chronic LCMV infection. Using ATAC-seq, it showed that the NBRE motif was highly enriched in opened chromatin regions associated with CD8^+^ T cell exhaustion (47). Similar observations were made in exhausted CD8^+^ tumor-infiltrating T lymphocytes (TILs) where the NBRE motif was enriched in open chromatin regions of Ag-specific CD8^+^ TILs when compared to bystander CD8^+^ TILs (48). The transcription of *Nr4a2* and *Nr4a3* was upregulated in CD8^+^ TILs in an autochthonous melanoma mouse model (97). Evidence for a similar involvement of NR4A in human exhausted CD8^+^ T cells came from a study of TILs in colorectal cancer where *Nr4a1* transcription and NBRE motif in transcriptionally active hypomethylated DNA regions were enriched in Ag-specific CD8^+^ TILs (CD39^+^CD103^+^) compared to bystander TILs (CD39^−^CD103^+^ or CD39^−^CD103^−^) (98).

The functional importance of NR4As in CD8^+^ T cell exhaustion during cancer was recently described (67, 88). Chen et al. demonstrated in mouse melanoma that there was substantial enrichment of NBRE motifs in the open chromatin regions of exhausted TILs and that the expression of NR4As is highly enriched in severely exhausted (PD-1^hi^ Tim-3^hi^) compared to exhausted (PD-1^hi^ Tim-3^lo^) CD8^+^ T cells (67). Similarly, the analysis of human melanoma TILs scRNA-Seq data highlighted a correlation of *Nr4a1*, *Nr4a2*, and *Nr4a3* transcription with inhibitory receptor expression (*Pdcd1* and *Havcr2*) (67). In addition to regulating the function of endogenous TIL, NR4As also regulated the function of chimeric antigen receptor (CAR) T cells. Adoptive T cell therapy (ACT) of B16 melanomas with CAR-T cells deficient for all three NR4A family members dramatically improved tumor control and survival over ACT with wild-type CAR-T cells (67). CAR TILs lacking NR4A1/2/3 expressed lower levels of the inhibitory receptors PD-1 and Tim-3 and produce more cytokines (TNF-α, IFN-γ) than their wild-type counterpart. In this setting, individual NR4A deletion did not confer any therapeutic effect suggesting redundant functions of the different NR4A members in CD8^+^ T cell exhaustion (67).

A similar role for NR4A1 in CD8^+^ T cell exhaustion was simultaneously reported by the group of Dong (88). In their study, the authors hypothesized a role for NR4A1 in CD8^+^ T cell exhaustion based on the observation that *Nr4a1* transcription is abolished and that the NBRE motif is lost in open chromatin regions in CD8^+^ TILs following anti-PD1 treatment, a treatment that reinvigorate exhausted T cells. They showed that ACT with *Nr4a1* ^−/−^ CD8^+^ T cells provide better tumor control than ACT with wild-type CD8^+^ T cells (88). This was associated with an increase in the number of CD8 TILs, reduction of PD-1 and Tim-3 expression, enhanced cytokine production (TNF-α and IFN-γ) as well as increased degranulation by CD8^+^ TILs (88). Similar observations were made using chronic infection with LCMV clone 13 further supporting an essential role for NR4A1 in programming CD8^+^ T cell exhaustion (88). This contrasts with the above CAR T cell model where the deletion of all three NR4A family members was required for therapeutic efficacy (67) and might result from more severe exhaustion in the CAR T cell system, which in turn requires the complete loss of NR4A activity.

Another recent study further supports the central role of NR4A family members in CD8^+^ T cell exhaustion. It was shown that NR4A and TOX transcription factors act downstream of NFAT to induce the transcriptional program of exhaustion. Furthermore, a positive feedback loop where both TOX and NR4A positively regulate each other is at play during CD8^+^ T cell exhaustion (99).

The role of NR4As in CD8^+^ T cell exhaustion (**Table 1**) suggests that manipulating their expression or developing drugs that modulate their activity represents a very promising strategy to prevent exhaustion during cancer immunotherapy treatment. Before doing so, it will be essential to determine whether NR4As act similarly in human T cells and, if so, to consider if inhibitors targeting these molecules have the undesired side-effect of enhancing autoimmunity.

## Molecular Mechanisms by Which NR4As Affect Acute and Chronic CD8^+^ T Cell Response

### Acute CD8^+^ T Cell Response

As discussed above, there is experimental evidence suggesting a role for NR4A1 and NR4A3 in the CD8^+^ T cell response to acute infection (68, 88, 89) with both members affecting the production of cytokines. Furthermore, NR4A1 and NR4A3 deficiency seems to have opposite effects on SLEC/MPEC differentiation and the three members were reported to have an impact on the memory generation (89, 94, 100). Very few studies have addressed the molecular events control by NR4As during CD8^+^ T cell response.

The group of Hedrick has reported that NR4A1 directly binds to the *Irf4* promoter, an event that leads to the inhibition of *Irf4* transcription (68). This transcriptional repression could be mediated *via* the demonstrated ability of NR4As in other settings to recruit the corepressor complex CoREST (43, 106) but a formal demonstration in CD8^+^ T cells is still lacking. In the absence of NR4A1, elevated *Irf4* transcription could explain the increased T cell expansion, cytokine production, and SLEC differentiation (107–111). Whether the phenotype of NR4A1-deficient T cells is solely the consequence of change in IRF4 expression levels needs further investigation. Both NR4A1 and NR4A3 deficiency led to better cytokine production, however, using RNA-seq we did not observe an increase in *Irf4* transcription by *Nr4a3* ^−/−^ CD8^+^ T cells (89), suggesting that other mechanisms are important in CD8^+^ T cells. To gain insight into the mechanism of NR4A3 action during acute CD8^+^ T cell response, we have used RNA-seq and ATAC-seq to identify the genes that are regulated by NR4A3. As NR4A3 is expressed very early following T cell activation, we performed these analyses at relatively early time points (12h after *in vitro* stimulation for ATAC-seq and *in vivo* day 3 post-infection for RNA-seq). The transcripts that are differentially expressed between *Nr4a3* ^+/+^ and *Nr4a3* ^−/−^ CD8^+^ T cells were associated with the signature of memory T cells. Furthermore, as early as day 3 post-infection, the expression of the transcription factors controlling MPEC differentiation (*Eomes*, *Tcf7*, *Id3*, *Bcl6*, *Bach2*, and *Zeb1*) is increased in absence of NR4A3 while the transcription of transcription factors involved in SLEC differentiation is reduced (*Tbx21*, *Prdm1*, *Id2, Rbpj*, and *Zeb2*), which explains why more MPECs and memory T cells are generated without NR4A3 (89). Further studies are needed to determine whether NR4A3 directly regulates the expression of the transcription factors controlling MPEC/SLEC differentiation as ATAC-seq analysis did not reveal differences in chromatin accessibility at the genes encoding these transcription factors, except for *Bach2* and *Rbpj* (89). Within the differentially accessible regions (DARs) that are less open in *Nr4a3* ^−/−^ CD8^+^ T cells, there was an expected enrichment for the NBRE motif, which suggests that several genes within these regions are direct targets of NR4A3. However, with the current knowledge, the list of genes within these regions did not help to explain how NR4A3 affects CD8^+^ T cell differentiation and function (89). Intriguingly, most of the DARs are more opened in absence of NR4A3 and within these regions there is an enrichment for the DNA binding motifs for the transcription factors of the bZIP family, which includes Fos and Jun (89). It is therefore possible that NR4A3 prevents the activity of bZIP transcription factors during CD8^+^ T cell response. As members of the bZIP transcription factor family such as AP-1 (Fos/Jun) and Bach2 are known to regulate cytokine production, these observations provide a mechanism for how NR4A3 influences T cell functions. However, whether the same mechanism contributes to enhance MPEC and central emory CD8^+^ T cell differentiation requires further investigation and it is unclear how NR4A3 prevents the accessibility to DNA of bZIP transcription factors. In CD4^+^ T cells, Liu et al. (discussed below) propose that NR4A1 directly compete with the binding of bZIP transcription factors to DNA (88). Combined with our ATAC-seq data in *Nr4a3^−/−^* CD8+ T cells, this suggests a common molecular mechanism used by different NR4A family members to influence gene transcription (**Figure 2B**). However, it remains unclear why NR4A1 and NR4A3 have different effects on MPEC/SLEC differentiation. Furthermore, how NR4A family members regulate CD8^+^ Trm differentiation at the molecular level is still unknown, but of critical importance.

### Chronic CD8^+^ T Cell Response

**Figure 3:**
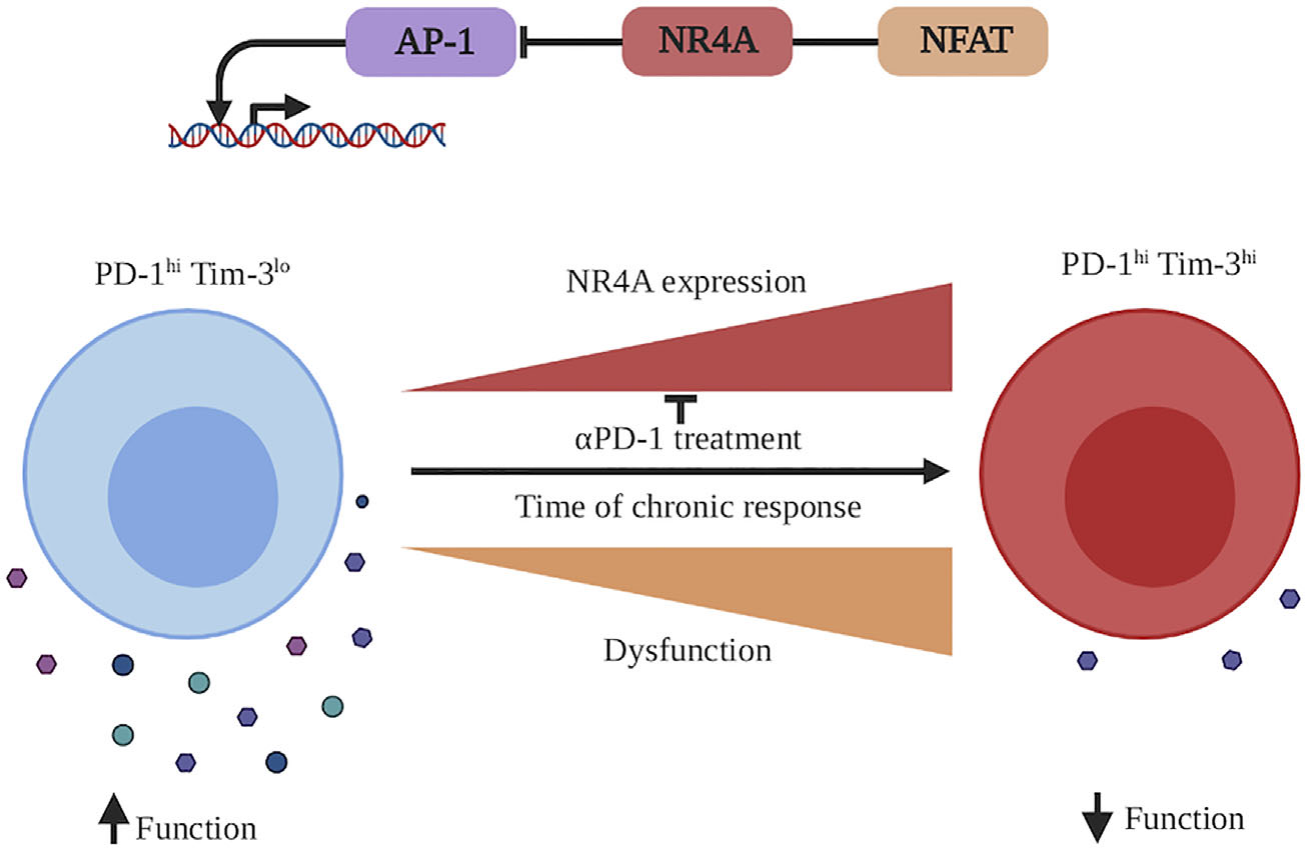
NR4A involvement in CD8+ T cell exhaustion during a chronic immune response. During a chronic immune response, antigen persistence as well as inflammation milieu will induce CD8^+^ T cell exhaustion. This state is characterized by the acquisition of the expression of different inhibitory receptors (as PD-1, Tim-3 etc.), which dampens CD8^+^ T cell function to protect the organism against the chronically activated CD8^+^ T cells. CD8^+^ T cell exhaustion is accompanied by an increased transcription of *Nr4a1, Nr4a2*, *and Nr4a3*. At the molecular level, NR4A family members cooperate with NFAT and potentially other transcription factors to decrease the activity of AP-1 (bZIP family transcription factor) and increase the dysfunctional/exhaustion state of the CD8+ T cells. An effective reinvigorating therapy to reverse CD8^+^ T cell dysfunction is treatment with anti-PD-1 antibodies, which decreases the *Nr4a* transcription. This figure was created with Biorender.com.

NR4A family members are important players in the induction of CD8^+^ T cell exhaustion (67, 88, 89). Transcriptomic analysis reveals that better tumor control by NR4A triple-deficient CD8^+^ T cells is associated with the induction of the effector T cells gene signature while those for exhausted and memory T cells were down-regulated (67). ATAC-seq further showed that a large fraction (36%) of DARs with lower accessibility in NR4A triple-deficient TILs contains the NBRE motif. A smaller fraction (11%) of DARs with lower accessibility contains a NFAT binding motif without an adjacent AP-1 site, a molecular pattern that is found within chromatin regions associated with exhaustion (47, 67). Interestingly, the DARs less accessible in NR4A triple-deficient TILs are very similar to those more open in CD8^+^ T cells expressing the exhaustion-inducing engineered form of NFAT that cannot interact with AP-1 (CA-RIT-NFAT) (47, 48, 96). Overall this suggests that NR4As directly contribute to the regulation of genes involved in CD8^+^ T cell exhaustion and explains why their deletion reduces T cell exhaustion. On the other hand, DARs that are more accessible in absence of NR4A members are enriched in the DNA binding motifs of bZIP (71%) and Rel/NFKB (25%) transcription factors (67). As these transcription factors have been reported to control T cell activation and effector functions, these molecular changes are probably responsible for the enhanced functionality that is observed in TILs deficient for NR4A family members (**Figure 3**). It is interesting to note that a similar impact on accessibility of chromatin regions containing bZIP transcription factor binding motifs was observed in CD8^+^ T cells lacking NR4A3 expression during response to acute *Listeria* infection and in CD4^+^ T cells lacking NR4A1 (88, 89). Limiting access of bZIP transcription factors to DNA is therefore a general mechanism of action of NR4As. Further studies should reveal whether each NR4A family member affects different sets of genes and/or influences different members of the bZIP transcription family.

## NR4As and Peripheral CD4^+^ T Cell Responses

CD4^+^ T cells act as central orchestrator of the adaptive immune response to pathogens and cancer. These cells help a variety of other immune cells, such as B cells, macrophages, and CD8^+^ T cells, to mount an immune response that is adapted to the type of infectious agents. Following Ag recognition of peptide fragments on MHC class II molecules on DCs, naïve CD4^+^ T cells will proliferate and differentiate into effector cells. Depending on the inflammatory context induced by the infecting pathogen, naïve CD4^+^ T cells can differentiate into several types of effectors: Th1, Th2, Th17, Tfh, or Treg. Briefly, Th1 cells are characterized by their ability to secrete IFN-γ, which will help to induce anti-microbial activity of macrophages and enhance CD8^+^ T cell response, Th2 production of IL-4 will help B cells to undergo class-switch to IgE, Th17 production of IL-17 will help recruit neutrophils, Tfh (T follicular helper) cells will help B cell responses and Treg cells will suppress the response of auto-reactive T cells. As for CD8^+^ T cells, after pathogen clearance most of the effectors will die while a few will further differentiate into long-lived CD4^+^ memory T cells.

In naïve CD4 T cells, NR4A1 is expressed at low level as a consequence of homeostatic/tonic TCR signaling (39)*. Nr4a* transcription is also rapidly induced in CD4^+^ T cells following engagement of the TCR by Ag. *In vitro*, this occurs within 1h of stimulation, peaks after 3–4h and return to basal level at 12h (38, 69, 112). The studies that have revealed some of the role of NR4A family members in CD4^+^ T-cell biology will now be discussed (**Table 2**).

**Table 2:** Role of NR4A family members in CD4 T cells.

Expression	*Nr4a1*	*Nr4a2*	*Nr4a3*	References
Rapid and transient induction by *in vitro* TCR stimulation	✓	✓	✓	(38, 69, 74, 87, 88, 90, 112)
Th1	Low	Low	Low	(113)
Th2	Low	Low	Low	(113)
Th17	Low	Low	Low	(113)
Tfh	✓ or low	✓ or low	Low	(112, 113)
T reg	✓	✓	✓	(113)
Dysfunctional/exhausted or tolerant CD4 T cells	✓or low	✓or low	✓	(88, 96, 97)
				
**Function**	**NR4A1**	**NR4A2**	**NR4A3**	**References**
T cell proliferation	ê	N.D.	N.D.	(69)
Cytokine production	ê	é	N.D.	(69, 88, 114)
Th1 polarization	ê	ê or no effect	N.D.	(69, 74, 88, 115)
Th2 polarization	No effect	N.D.	N.D.	(88)
Th17 polarization	ê	ê or é	N.D.	(69, 74, 88, 115)
iTreg differentiation Treg identity	No effect é	é é	N.D. é	(74, 88) (113)
Tfh development and function *in vivo*	No effect in TKO	No effect in TKO	No effect in TKO	(112)
T cell tolerance	é	N.D.	N.D.	(88)
				
**Molecular mechanism**	**NR4A1**	**NR4A2**	**NR4A3**	**References**
Competition for bZIP transcription binding activity	✓	N.D.	N.D.	(88)

The transcription of *Nr4a2* is highly enriched in peripheral blood T cells of multiple sclerosis (MS) patients and in T cells during experimental autoimmune encephalomyelitis (EAE), a mouse model of MS (114, 116). The overexpression of NR4A2 in primary mouse T cells increased the production of IFN-γ and IL-17, the main cytokines involved in MS/EAE pathogenesis. Conversely, its suppression decreased IFN-γ and IL-17 production and the induction of EAE was reduced following the adoptive transfer of encephalitogenic CD4^+^ T cells in which NR4A2 expression was reduced using siRNA when compared to control CD4^+^ T cells (114). Importantly, siRNA-mediated knockdown of NR4A2 in CD4^+^ T cells from MS patients also led to reduced IFN-γ and IL-17 production (114). A luciferase promoter assay suggests that NR4A2 acts directly on the transcription of *Ifng* and *Il17* genes (114). A follow-up study by the same group, showed *Nr4a2* transcription was selectively higher in IL-17- or IL-17/IFN-γ-producing CD4^+^ T cells when compared to IFN-γ-producing CD4^+^ T cells during EAE and experimental autoimmune uveitis (EAU) (115). This increase in NR4A2 expression by autoimmune T cells was not observed in the STZ model of autoimmune diabetes, which is mediated by Th1 cells, suggesting that the enhanced expression of NR4A2 is associated with autoimmune diseases where IL-17 plays a pathogenic role (115). To understand the role of NR4A2 induction in IL-17 production by CD4^+^ T cells the authors used siRNA knockdown of *Nr4a2* and showed that NR4A2 decreases *in vitro* Th17 differentiation but not Th1 differentiation. The effect on Th17 differentiation was not due to decreased expression of RORγt, the master transcription factor controlling Th17 differentiation. Instead, NR4A2 was necessary for the production of IL-21, which then upregulates the expression of the IL-23 receptor, necessary to enhance and stabilize the Th17 phenotype. Furthermore, the addition of IL-21 rescued Th17 differentiation by *Nr4a2* knockdown CD4^+^ T cells (115). Injection of mice, early or late during EAE, with siRNA directed against *Nr4a2* was able to significantly reduce EAE clinical scores with a concomitant decrease of IL-17, but not IFN-γ, production by CD4^+^ T cells that have infiltrated the central nervous system (115). Therefore, NR4A2 expression in CD4^+^ T cells promotes Th17 differentiation and targeting its expression represents a promising strategy to treat MS patients. It is intriguing that NR4A2 promotes cytokine production by CD4^+^ T cells since NR4A1 and NR4A3 were shown to dampen cytokine production by CD8^+^ T cells. As the three family members recognize the same motifs on DNA, further studies are required to determine whether this is the consequence of a different function of NR4A2 or a cell type specific effect.

Although NR4A expression is transiently induced following CD4^+^ T cell activation, it was reported that Tfh cells transcribe *Nr4a1* and *Nr4a2* (112). This is probably the consequence of the continuous TCR stimulation of Tfh cells by antigen-presenting cognate B cells within the germinal centers. However, the deletion of the three family members in CD4^+^ T cells did not affect Tfh differentiation and function (112).

The role of the NR4A family members in CD4^+^ T cell response was recently broadened by the identification of the involvement of NR4A1 in CD4^+^ T cell activation, metabolism, tolerance, and autoimmunity (69, 88). Liebmann et al. demonstrated that NR4A1 deletion in CD4^+^ T cells enhances T cell proliferation and cytokine production both *in vitro* and *in vivo* (69). The deletion of *Nr4a1* in the 2D2 TCR transgenic mouse model of EAE led to accelerated and more severe disease with an increase in IFN-γ and IL-17 secreting CD4^+^ T cells within the central nervous system. The authors further confirmed that it was the lack of NR4A1 in T cells that was involved using adoptive T cell transfer experiments (69). They also showed a general role for NR4A1 in different T cell mediated inflammatory diseases such as allergic contact dermatis and collagen-induced arthritis (69). Increased proliferation in absence of NR4A1 was not the consequence of reduced apoptosis but correlated with an increase in cell cycle entry. As entry into the cell cycle is regulated by metabolism, the authors evaluated whether NR4A1 deficiency impacted T cell metabolism. In absence of NR4A1, activated CD4^+^ T cells showed increased respiration, glycolysis, and glycolytic activity. As a consequence, the pharmacological inhibition of respiration or glycolysis had much less effect on proliferation of *Nr4a1* ^−/−^ than *Nr4a1* ^+/+^ CD4^+^ T cells (69). In agreement with a role for NR4A1 in regulating T cell metabolism was the regulation of several genes involved in T cell metabolism such as electron transport genes and genes controlling glucose metabolism. Intriguingly, the analysis of motifs within the promoters of metabolic genes that are differentially expressed between *Nr4a1* ^−/−^ and *Nr4a1* ^+/+^ activated CD4^+^ T cells did not reveal NR4A1 as a possible upstream regulator but predicted a role for the nuclear receptors ERRα, ERRγ, ERRβ, NR2F1, and NR0B1. Furthermore, NR4A1 was shown to bind to the *Esrra* gene, encoding for ERRα. The authors demonstrated that pharmacological inhibition of ERRα or shRNA knockdown of *Esrra* partially reversed the phenotype (cytokine production and metabolism) of NR4A1-deficient CD4^+^ T cells and reduced EAE disease severity of *Nr4a1* ^−/−^ mice (69). This highlights the key role of NR4A1 transcriptional induction of *Esrra*. Further studies should reveal which other mechanisms contribute to the CD4^+^ T cell phenotype in absence of NR4A1 and whether other family members regulate T cell metabolism.

A pivotal role of NR4A1 in CD4^+^ T cell dysfunction was recently described by the group of Dong (88). The authors observed a specific upregulation of NR4A1 in CD4^+^ tolerant T cells. The overexpression of NR4A1 in CD4^+^ T cells strongly suppressed the expression of genes associated with effector functions while inducing the expression of anergy-related genes following TCR stimulation. Under Th polarizing culture conditions, overexpression of NR4A1 impaired Th1 and Th17 differentiation without affecting Treg and Th2 generation. On the other hand, the inactivation of *Nr4a1* in CD4^+^ T cells enhanced IL-2 and IFN-γ production. This suggests that NR4A1 is overexpressed in CD4^+^ tolerant T cells precisely to induce tolerance. This was tested *in vivo* using an oral tolerance model where *Nr4a1* deletion increased IL-2 and IFN-γ production and prevented the establishment of CD4^+^ T cell tolerance. Further supporting the role for NR4A1 in repressing effector functions of CD4^+^ T cells, the adoptive transfer of naïve *Nr4a1* ^−/−^ CD4^+^ T cells into RAG-deficient mice induced more severe colitis than wild-type CD4^+^ T cells, with an increase in IFN-γ and IL-17 producing T cells in the colon (88). The comparison of the transcriptome of NR4A1 overexpressing CD4^+^ T cells and CD4^+^ tolerant T cells revealed a common gene signature containing a core cluster of genes controlling T cell activation or dysfunction. A ChIP-seq experiment revealed that approximately 70% of the CD4^+^ tolerance T cells genes were direct targets of NR4A1. Further analysis of the ChIP-seq data unexpectedly identified AP-1 consensus sequences and canonical NBRE motifs at sites where NR4A1 bound. This suggests that NR4A1 might be able to compete with AP-1 (bZIP) family members for overlapping sites on DNA and would explain the fact that most of the NR4A1 target genes that were downregulated by NR4A1 overexpression had reduced c-Jun binding. Inhibition of AP-1 activity by NR4A1 was demonstrated using luciferase reporter and EMSA assays suggesting that after being recruited to AP-1 binding sites, NR4A1 can repress effector gene expression. In addition, for the genes bound by NR4A1 and whose transcription is increased by NR4A1, these largely co-localized with H3K27 acetylation marks and include genes involved in tolerance. In summary, NR4A1 modulates the transcriptional program of CD4^+^ T cells by directly upregulating the expression of target genes containing NBRE motifs while downregulating the expression of AP-1 target genes (88). Interestingly, the repression of AP-1 target genes by NR4As is important in CD8^+^ T cells (67, 89) indicating that this is general mechanism by which they repress effector functions in T cells. Whether NR4A members solely influences the activity of AP-1 requires future investigation as other transcription factor binding motifs, such as NFKB, were identified in open chromatin regions of NR4A-deficient CD8^+^ T cells (67, 89). In agreement with this possibility is an older study performed in the Jurkat T cell lines showing that NR4A1 can compete with NFKB for binding to DNA (117).

NR4A-family members do not only have a role in the development of regulatory CD4^+^ T cells, as discussed above, but they are also required to maintain a pool of fully functional Tregs. NR4A1, NR4A2 and NR4A3 protein and transcript levels in peripheral Treg largely exceed those measured in other mature T cell subsets (42, 113). To evaluate the importance of NR4As in more mature Treg cells, specific deletion of *Nr4a1* and *Nr4a2* in this subset was obtained using a *Foxp3* Cre/Lox system (113). This was combined to a germline *Nr4a3* deletion to generate *Nr4a* triple knockout (TKO) in Foxp3 expressing cells. While generated efficiently in the thymus, Treg cells from these mice have a competitive disadvantage in the periphery (113). In addition, TKO Treg cells have decreased Foxp3 expression, lose their suppressive functions and gain Tfh and Th2 gene expression programs (113). This is because, as demonstrated by NR4A1 ChIP-seq, NR4As directly bind and maintain active chromatin marks on Treg-associated genes *Foxp3, Il2ra*, and *Ikzf4*. In addition, specifically in Tregs, NR4A1 directly binds and represses the *Il4* and *Il21* loci. A luciferase reporter assay also demonstrated that NR4A2 suppresses *Il4* promoter activity. Therefore, in mature CD4^+^ Treg cells, NR4As serve to maintain regulatory identity while suppressing Th2 and Tfh programs. The different NR4A family members vary in their capacity to induce Foxp3 expression (74) and the fact that the effects on Treg function was, at least partially, reproduced in single *Nr4a2* deficient mice suggests that perhaps NR4A2 could be the main driver of Treg identity in mature CD4 T cells (74). However this is in slight contradiction with the fact that autoimmunity and reduced lifespan was observed in *Nr4a1*/*Nr4a3* but not in *Nr4a2*/*Nr4a3* double deficient mice (113). Finally, conditional acute deletion of *Nr4a1* and/or *Nr4a2* in ERT2-Cre mice resulted in the loss of Treg-associated transcriptional targets and inhibited the *in vitro* differentiation of inducible Treg cells (74, 113). It is thus unlikely that the effects observed in TKO mice generated with the Foxp3-Cre system are solely the consequence of poorly matured Foxp3^+^ thymocytes (74). The dependence of Treg cells on NR4A expression makes it a possible target for therapy. For example, in the tumor microenvironment, regulatory T cells are detrimental and are associated with poor prognosis (118–120). Treg-specific *Nr4a1* and *Nr4a2* deficient mice have increased tumor resistance and pharmacological treatments that inhibit the expression of these NR4As in tumoral Treg cells result in improved CD8^+^ T cell functions and tumor control (121).

The early induction of NR4A in CD4^+^ T cells by TCR signaling influences proliferation, metabolism, function, and differentiation of conventional CD4^+^ T cells. Interestingly, the deletion of NR4A1 or NR4A2 seems to have different impact on T cell functions where NR4A1 suppress effector gene expression while NR4A2 positively contributes to the expression of cytokine genes and Th17 polarization. As a consequence suppressing NR4A1 expression promotes autoimmunity while deletion of NR4A2 protects from Th17-mediated autoimmune diseases. Furthermore, NR4A1 contributes to the induction of the tolerance program in CD4^+^ T cells. Therefore, targeting of a specific family member will offer unique therapeutic opportunity to either enhance or inhibit CD4^+^ T cell response. Furthermore, NR4As play important role in the maintenance of Treg cell identity. Future studies are needed to evaluate whether NR4A3 contributes to CD4^+^ T cell response and whether any member influences memory CD4^+^ T cell development. Altogether, this underlies the importance of teasing apart the unique role of each of NR4A family member in CD4^+^ T cells as this information will be key for being specifically able to appropriately target CD4-mediated autoimmune/inflammatory diseases, to enhance anti-tumor response and to therapeutically induce tolerance.

## Concluding Remarks

In T-cell biology, NR4A1, NR4A2, and NR4A3 have long been considered as functionally redundant. While this certainly appears to be true to some extent, there is also significant evidence that shows that, as a function of the model, cell type, or the measured output, these molecules have some unique roles. Given the important therapeutic roles NR4As could play in autoimmunity, vaccination, or cancer, it is exciting to think that there is still significant work left to understand their common and distinctive molecular modes of action.

## Author Contributions

LO, JM, SB, TB, and NL wrote the manuscript and prepared the figures. TB and NL edited the manuscript. LO and JM contributed equally to the work. All authors contributed to the article and approved the submitted version.

## Funding

This work was supported by grants from the Canadian Institutes of Health Research (MOP 142333 and PJT 168910) and by a discovery grant from the Natural Sciences and Engineering Research Council of Canada (RGPIN-2015-06645) to NL and by a Canadian Institutes of Health Research (PS 156104) to TB. LO was supported by studentships from the Fonds de la Recherche Québec-Santé and from the Cole Foundation. JM was supported by the Dean’s Doctoral Award from the Faculty of Medicine and Dentistry, University of Alberta.

## Conflict of Interest

The authors declare that the research was conducted in the absence of any commercial or financial relationships that could be construed as a potential conflict of interest.

## References

[1] Philips A Lesage S Gingras R Maira MH Gauthier Y Hugo P Novel dimeric Nur77 signaling mechanism in endocrine and lymphoid cells. Mol Cell Biol (1997) 17:5946–51. 10.1128/MCB.17.10.5946 PMC232442 9315652

[2] Winoto A Littman DR Nuclear hormone receptors in T lymphocytes. Cell (2002) 109 Suppl:S57–66. 10.1016/S0092-8674(02)00710-9 11983153

[3] Pearen MA Muscat GEO Minireview: Nuclear hormone receptor 4A signaling: implications for metabolic disease. Mol Endocrinol (2010) 24:1891–903. 10.1210/me.2010-0015 PMC5417389 20392876

[4] Herring JA Elison WS Tessem JS Function of Nr4a Orphan Nuclear Receptors in Proliferation, Apoptosis and Fuel Utilization Across Tissues. Cells (2019) 8:1373. 10.1210/me.2007-0464 PMC6912296 31683815

[5] Hogquist KA Baldwin TA Jameson SC Central tolerance: learning self-control in the thymus. Nat Rev Immunol (2005) 5:772–82. 10.1038/nri1707 16200080

[6] Klein L Kyewski B Allen PM Hogquist KA Positive and negative selection of the T cell repertoire: what thymocytes see (and don’t see). Nat Rev Immunol (2014) 14:377–91. 10.1038/nri3667 PMC4757912 24830344

[7] Hogquist KA Jameson SC The self-obsession of T cells: how TCR signaling thresholds affect fate “decisions” and effector function. Nat Immunol (2014) 15:815–23. 10.1038/ni.2938 PMC4348363 25137456

[8] Anderson MS Venanzi ES Klein L Chen Z Berzins SP Turley SJ Projection of an immunological self shadow within the thymus by the aire protein. Science (2002) 298:1395–401. 10.1126/science.1075958 12376594

[9] Takaba H Morishita Y Tomofuji Y Danks L Nitta T Komatsu N Fezf2 Orchestrates a Thymic Program of Self- Antigen Expression for Immune Tolerance. Cell (2015) 163:975–87. 10.1016/j.cell.2015.10.013 26544942

[10] Baldwin TA Hogquist KA Transcriptional analysis of clonal deletion in vivo. J Immunol (2007) 179:837–44. 10.4049/jimmunol.179.2.837 17617574

[11] Xing Y Hogquist KA T-Cell Tolerance: Central and Peripheral. Cold Spring Harb Perspect Biol (2012) 4:a006957–a006957. 10.1101/cshperspect.a006957 22661634 PMC3367546

[12] Cheng LE Chan FK Cado D Winoto A Functional redundancy of the Nur77 and Nor-1 orphan steroid receptors in T-cell apoptosis. EMBO J (1997) 16:1865–75. 10.1093/emboj/16.8.1865 PMC1169790 9155013

[13] Woronicz JD Calnan B Ngo V Winoto A Requirement for the orphan steroid receptor Nur77 in apoptosis of T-cell hybridomas. Nature (1994) 367:277–81. 10.1038/367277a0 8121493

[14] Liu ZG Smith SW McLaughlin KA Schwartz LM Osborne BA Apoptotic signals delivered through the T-cell receptor of a T-cell hybrid require the immediate-early gene nur77. Nature (1994) 367:281–4. 10.1038/367281a0 8121494

[15] Jennings E Elliot TAE Thawait N Kanabar S Yam-Puc JC Ono M Nr4a1 and Nr4a3 Reporter Mice Are Differentially Sensitive to T Cell Receptor Signal Strength and Duration. Cell Rep (2020) 33:108328. 10.1016/j.celrep.2020.108328 33147449 PMC7653457

[16] Sohn SJ Thompson J Winoto A Apoptosis during negative selection of autoreactive thymocytes. Curr Opin Immunol (2007) 19:510–5. 10.1016/j.coi.2007.06.001 17656079

[17] Won HY Hwang ES Transcriptional modulation of regulatory T cell development by novel regulators NR4As. Arch Pharm Res (2016) 11:1–7. 10.1007/s12272-016-0803-z 27778276

[18] Hu QN Baldwin TA Differential Roles for Bim and Nur77 in Thymocyte Clonal Deletion Induced by Ubiquitous Self-Antigen. J Immunol (2015) 194:2643–53. 10.4049/jimmunol.1400030 25687757

[19] Hu QN Suen AYW Henao Caviedes LM Baldwin TA Nur77 Regulates Nondeletional Mechanisms of Tolerance in T Cells. J Immunol (2017) 199:3147–57. 10.4049/jimmunol.1701085 28947542

[20] Li H Kolluri SK Gu J Dawson MI Cao X Hobbs PD Cytochrome c release and apoptosis induced by mitochondrial targeting of nuclear orphan receptor TR3. Science (2000) 289:1159–64. 10.1126/science.289.5482.1159 10947977

[21] Thompson J Winoto A During negative selection, Nur77 family proteins translocate to mitochondria where they associate with Bcl-2 and expose its proapoptotic BH3 domain. J Exp Med (2008) 205:1029–36. 10.1084/jem.20080101 PMC2373836 18443228

[22] Lin B Kolluri SK Lin F Liu W Han Y-H Cao X Conversion of Bcl-2 from protector to killer by interaction with nuclear orphan receptor Nur77/TR3. Cell (2004) 116:527–40. 10.1016/s0092-8674(04)00162-x 14980220

[23] Wang A Rud J Olson CM Anguita J Osborne BA Phosphorylation of Nur77 by the MEK-ERK-RSK cascade induces mitochondrial translocation and apoptosis in T cells. J Immunol (2009) 183:3268–77. 10.4049/jimmunol.0900894 19675165

[24] Ohkura N Hijikuro M Yamamoto A Miki K Molecular cloning of a novel thyroid/steroid receptor superfamily gene from cultured rat neuronal cells. Biochem Biophys Res Commun (1994) 205:1959–65. 10.1006/bbrc.1994.2900 7811288

[25] Law SW Conneely OM DeMayo FJ O’Malley BW Identification of a new brain-specific transcription factor, NURR1. Mol Endocrinol (1992) 6:2129–35. 10.1210/mend.6.12.1491694 1491694

[26] Woronicz JD Lina A Calnan BJ Szychowski S Cheng L Winoto A Regulation of the Nur77 orphan steroid receptor in activation-induced apoptosis. Mol Cell Biol (1995) 15:6364–76. 10.1128/mcb.15.11.6364 PMC230888 7565789

[27] Calnan BJ Szychowski S Chan FK Cado D Winoto A A role for the orphan steroid receptor Nur77 in apoptosis accompanying antigen-induced negative selection. Immunity (1995) 3:273–82. 10.1016/1074-7613(95)90113-2 7552993

[28] Lee SL Wesselschmidt RL Linette GP Kanagawa O Russell JH Milbrandt J Unimpaired thymic and peripheral T cell death in mice lacking the nuclear receptor NGFI-B (Nur77). Science (1995) 269:532–5. 10.1126/science.7624775 7624775

[29] Dhein J Walczak H Bäumler C Debatin KM Krammer PH Autocrine T-cell suicide mediated by APO-1/(Fas/CD95). Nature (1995) 373:438–41. 10.1038/373438a0 7530335

[30] Zhou T Cheng J Yang P Wang Z Liu C Su X Inhibition of Nur77/Nurr1 leads to inefficient clonal deletion of self-reactive T cells. J Exp Med (1996) 183:1879–92. 10.1084/jem.183.4.1879 PMC2192482 8666944

[31] Kisielow P Blüthmann H Staerz UD Steinmetz M Boehmer von H Tolerance in T-cell-receptor transgenic mice involves deletion of nonmature CD4+8+ thymocytes. Nature (1988) 333:742–6. 10.1038/333742a0 3260350

[32] Mamalaki C Elliott J Norton T Yannoutsos N Townsend AR Chandler P Positive and negative selection in transgenic mice expressing a T-cell receptor specific for influenza nucleoprotein and endogenous superantigen. Dev Immunol (1993) 3:159–74. 10.1155/1993/98015 PMC2275926 8281031

[33] Dequiedt F Kasler H Fischle W Kiermer V Weinstein M Herndier BG HDAC7, a thymus-specific class II histone deacetylase, regulates Nur77 transcription and TCR-mediated apoptosis. Immunity (2003) 18:687–98. 10.1016/s1074-7613(03)00109-2 12753745

[34] Oh S Oh J Lee C Oh S Jeon S Choi J Expression of Twist2 is controlled by T-cell receptor signaling and determines the survival and death of thymocytes. Cell Death Differ (2016) 23:1804–14. 10.1038/cdd.2016.68 PMC5071571 27391798

[35] Dequiedt F Van Lint J Lecomte E Van Duppen V Seufferlein T Vandenheede JR Phosphorylation of histone deacetylase 7 by protein kinase D mediates T cell receptor–induced Nur77 expression and apoptosis. J Exp Med (2005) 201:793–804. 10.1016/S1097-2765(01)00429-4 15738054 PMC2212830

[36] Parra M Kasler H McKinsey TA Olson EN Verdin E Protein Kinase D1 Phosphorylates HDAC7 and Induces Its Nuclear Export after T-cell Receptor Activation. J Biol Chem (2005) 280:13762–70. 10.1046/j.1365-2567.2000.00110.x 15623513

[37] Kasler HG Lim HW Mottet D Collins AM Lee IS Verdin E Nuclear export of histone deacetylase 7 during thymic selection is required for immune self-tolerance. EMBO J (2012) 31:4453–65. 10.1038/emboj.2012.295 PMC3512390 23103766

[38] Cunningham NR Artim SC Fornadel CM Sellars MC Edmonson SG Scott G Immature CD4+CD8+ thymocytes and mature T cells regulate Nur77 distinctly in response to TCR stimulation. J Immunol (2006) 177:6660–6. 10.4049/jimmunol.177.10.6660 17082578

[39] Kuang AA Cado D Winoto A Nur77 transcription activity correlates with its apoptotic function in vivo. Eur J Immunol (1999) 29:3722–8. 10.1002/(SICI)1521-4141(199911)29:11<3722::AID-IMMU3722>3.0.CO;2-N 10556828

[40] Rajpal A Cho YA Yelent B Koza-Taylor PH Li D Chen E Transcriptional activation of known and novel apoptotic pathways by Nur77 orphan steroid receptor. EMBO J (2003) 22:6526–36. 10.1093/emboj/cdg620 PMC291815 14657025

[41] Weih F Ryseck RP Chen L Bravo R Apoptosis of nur77/N10-transgenic thymocytes involves the Fas/Fas ligand pathway. Proc Natl Acad Sci USA (1996) 93:5533–8. 10.1073/pnas.93.11.5533 PMC39281 8643610

[42] Moran AE Holzapfel KL Xing Y Cunningham NR Maltzman JS Punt J T cell receptor signal strength in Treg and iNKT cell development demonstrated by a novel fluorescent reporter mouse. J Exp Med (2011) 208:1279–89. 10.1126/science.1103440 PMC3173240 21606508

[43] Nowyhed HN Huynh TR Blatchley A Wu R Thomas GD Hedrick CC The nuclear receptor nr4a1 controls CD8 T cell development through transcriptional suppression of runx3. Sci Rep (2015) 5:9059. 10.1038/srep09059 25762306 PMC4356985

[44] Baldwin TA Sandau MM Jameson SC Hogquist KA The timing of TCR alpha expression critically influences T cell development and selection. J Exp Med (2005) 202:111–21. 10.1084/jem.20050359 PMC2212895 15998791

[45] Sekiya T Kashiwagi I Yoshida R Fukaya T Morita R Kimura A Nr4a receptors are essential for thymic regulatory T cell development and immune homeostasis. Nat Immunol (2013) 14:230–7. 10.1038/ni.2520 23334790

[46] Fassett MS Jiang W D’Alise AM Mathis D Benoist C Nuclear receptor Nr4a1 modulates both regulatory T-cell (Treg) differentiation and clonal deletion. Proc Natl Acad Sci USA (2012) 109:3891–6. 10.1073/pnas.1200090109 PMC3309794 22345564

[47] Scott-Browne JP López-Moyado IF Trifari S Wong V Chavez L Rao A Dynamic Changes in Chromatin Accessibility Occur in CD8+ T Cells Responding to Viral Infection. Immunity (2016) 45:1327–40. 10.1016/j.immuni.2016.10.028 PMC5214519 27939672

[48] Mognol GP Spreafico R Wong V Scott-Browne JP Togher S Hoffmann A Exhaustion-associated regulatory regions in CD8+ tumor-infiltrating T cells. Proc Natl Acad Sci USA (2017) 114:E2776–85. 10.1073/pnas.1620498114 PMC5380094 28283662

[49] Huo J Xu S Lam K-P ASK1 Mediates Nur77 Expression in T-Cell Receptor Mediated Thymocyte Apoptosis. Cells (2020) 9:585 10.3390/cells9030585 PMC7140521 32121597

[50] Sohn SJ Lewis GM Winoto A Non-redundant function of the MEK5-ERK5 pathway in thymocyte apoptosis. EMBO J (2008) 27:1896–906. 10.1038/emboj.2008.114 PMC2486421 18548009

[51] Liston A Lesage S Wilson J Peltonen L Goodnow CC Aire regulates negative selection of organ-specific T cells. Nat Immunol (2003) 4:350–4. 10.1038/ni906 12612579

[52] Barnden MJ Allison J Heath WR Carbone FR Defective TCR expression in transgenic mice constructed using cDNA-based alpha- and beta-chain genes under the control of heterologous regulatory elements. Immunol Cell Biol (1998) 76:34–40. 10.1046/j.1440-1711.1998.00709.x 9553774

[53] Kurts C Heath WR Carbone FR Allison J Miller JF Kosaka H Constitutive class I-restricted exogenous presentation of self antigens in vivo. J Exp Med (1996) 184:923–30. 10.1084/jem.184.3.923 PMC2192761 9064352

[54] Bouillet P Purton JF Godfrey DI Zhang L-C Coultas L Puthalakath H BH3-only Bcl-2 family member Bim is required for apoptosis of autoreactive thymocytes. Nature (2002) 415:922–6. 10.1038/415922a 11859372

[55] Hogquist KA Jameson SC Heath WR Howard JL Bevan MJ Carbone FR T cell receptor antagonist peptides induce positive selection. Cell (1994) 76:17–27. 10.1016/0092-8674(94)90169-4 8287475

[56] Wingate AD Campbell DG Peggie M Arthur JSC Nur77 is phosphorylated in cells by RSK in response to mitogenic stimulation. Biochem J (2006) 393:715–24. 10.1042/BJ20050967 PMC1360724 16223362

[57] Masuyama N Oishi K Mori Y Ueno T Takahama Y Gotoh Y Akt inhibits the orphan nuclear receptor Nur77 and T-cell apoptosis. J Biol Chem (2001) 276:32799–805. 10.1074/jbc.M105431200 11438550

[58] Pekarsky Y Hallas C Palamarchuk A Koval A Bullrich F Hirata Y Akt phosphorylates and regulates the orphan nuclear receptor Nur77. Proc Natl Acad Sci USA (2001) 98:3690–4. 10.1073/pnas.051003198 PMC31113 11274386

[59] Thompson J Burger ML Whang H Winoto A Protein kinase C regulates mitochondrial targeting of Nur77 and its family member Nor-1 in thymocytes undergoing apoptosis. Eur J Immunol (2010) 40:2041–9. 10.1002/eji.200940231 PMC3076209 20411565

[60] Han Y-H Cao X Lin B Lin F Kolluri SK Stebbins J Regulation of Nur77 nuclear export by c-Jun N-terminal kinase and Akt. Oncogene (2006) 25:2974–86. 10.1038/sj.onc.1209358 16434970

[61] Liu B Wu J-F Zhan Y-Y Chen H-Z Zhang X-Y Wu Q Regulation of the Orphan Receptor TR3 Nuclear Functions by c-Jun N Terminal Kinase Phosphorylation. Endocrinology (2007) 148:34–44. 10.1210/en.2006-0800 17023523

[62] Gray DHD Kupresanin F Berzins SP Herold MJ O’Reilly LA Bouillet P The BH3-Only Proteins Bim and Puma Cooperate to Impose Deletional Tolerance of Organ-Specific Antigens. Immunity (2012) 37:451–62. 10.1016/j.immuni.2012.05.030 PMC3500635 22960223

[63] Kasler HG Victoria J Duramad O Winoto A ERK5 is a novel type of mitogen-activated protein kinase containing a transcriptional activation domain. Mol Cell Biol (2000) 20:8382–9. 10.1128/mcb.20.22.8382-8389.2000 PMC102145 11046135

[64] Fujii Y Matsuda S Takayama G Koyasu S ERK5 is involved in TCR-induced apoptosis through the modification of Nur77. Genes Cells (2008) 13:411–9. 10.1111/j.1365-2443.2008.01177.x 18429814

[65] Xue L Chiang L Kang C Winoto A The role of the PI3K-AKT kinase pathway in T-cell development beyond the beta checkpoint. Eur J Immunol (2008) 38:3200–7. 10.1002/eji.200838614 PMC2614442 18991293

[66] Huo J Xu S Lam K-P Fas apoptosis inhibitory molecule regulates T cell receptor-mediated apoptosis of thymocytes by modulating Akt activation and Nur77 expression. J Biol Chem (2010) 285:11827–35. 10.1074/jbc.M109.072744 PMC2852919 20178987

[67] Chen J López-Moyado IF Seo H Lio C-WJ Hempleman LJ Sekiya T NR4A transcription factors limit CAR T cell function in solid tumours. Nature (2019) 567:530–4. 10.1038/s41586-019-0985-x PMC6546093 30814732

[68] Nowyhed HN Huynh TR Thomas GD Blatchley A Hedrick CC Cutting Edge: The Orphan Nuclear Receptor Nr4a1 Regulates CD8+ T Cell Expansion and Effector Function through Direct Repression of Irf4. J Immunol (2015) 195:3515–9. 10.4049/jimmunol.1403027 PMC4592102 26363057

[69] Liebmann M Hucke S Koch K Eschborn M Ghelman J Chasan AI Nur77 serves as a molecular brake of the metabolic switch during T cell activation to restrict autoimmunity. Proc Natl Acad Sci USA (2018) 115:E8017–26. 10.1073/pnas.1721049115 PMC6112725 30072431

[70] Myers DR Lau T Markegard E Lim HW Kasler H Zhu M Tonic LAT-HDAC7 Signals Sustain Nur77 and Irf4 Expression to Tune Naive CD4 T Cells. Cell Rep (2017) 19:1558–71. 10.1016/j.celrep.2017.04.076 PMC5587137 28538176

[71] Veillette A Bookman MA Horak EM Bolen JB The CD4 and CD8 T cell surface antigens are associated with the internal membrane tyrosine-protein kinase p56lck. Cell (1988) 55:301–8. 10.1016/0092-8674(88)90053-0 3262426

[72] Legname G Seddon B Lovatt M Tomlinson P Sarner N Tolaini M Inducible expression of a p56Lck transgene reveals a central role for Lck in the differentiation of CD4 SP thymocytes. Immunity (2000) 12:537–46. 10.1016/s1074-7613(00)80205-8 10843386

[73] Kappes DJ He X He X CD4-CD8 lineage commitment: an inside view. Nat Immunol (2005) 6:761–6. 10.1016/S1074-7613(04)00109-8 16034433

[74] Sekiya T Kashiwagi I Inoue N Morita R Hori S Waldmann H The nuclear orphan receptor Nr4a2 induces Foxp3 and regulates differentiation of CD4+ T cells. Nat Commun (2011) 2:269. 10.1038/ncomms1272 21468021 PMC3104557

[75] Sekiya T Hibino S Saeki K Kanamori M Takaki S Yoshimura A Nr4a Receptors Regulate Development and Death of Labile Treg Precursors to Prevent Generation of Pathogenic Self-Reactive Cells. Cell Rep (2018) 24:1627–1638.e6. 10.1016/j.celrep.2018.07.008 30089271

[76] Ramsdell F Lantz T Fowlkes BJ A nondeletional mechanism of thymic self tolerance. Science (1989) 246:1038–41. 10.1126/science.2511629 2511629

[77] Martinez RJ Zhang N Thomas SR Nandiwada SL Jenkins MK Binstadt BA Arthritogenic self-reactive CD4+ T cells acquire an FR4hiCD73hi anergic state in the presence of Foxp3+ regulatory T cells. J Immunol (2012) 188:170–81. 10.4049/jimmunol.1101311 PMC3244540 22124124

[78] Kalekar LA Schmiel SE Nandiwada SL Lam WY Barsness LO Zhang N CD4(+) T cell anergy prevents autoimmunity and generates regulatory T cell precursors. Nat Immunol (2016) 17:304–14. 10.1038/ni.3331 PMC4755884 26829766

[79] Lacorazza HD Tuček-Szabo C Vasović LV Remus K Nikolich-Žugich J Premature TCRαβ Expression and Signaling in Early Thymocytes Impair Thymocyte Expansion and Partially Block Their Development. J Immunol (2001) 166:3184–93. 10.1016/S1074-7613(00)80576-2 11207271

[80] Takahama Y Shores EW Singer A Negative selection of precursor thymocytes before their differentiation into CD4+CD8+ cells. Science (1992) 258:653–6. 10.1126/science.1357752 1357752

[81] Koenis DS Medzikovic L Vos M Beldman TJ van Loenen PB van Tiel CM Nur77 variants solely comprising the amino-terminal domain activate hypoxia-inducible factor-1α and affect bone marrow homeostasis in mice and humans. J Biol Chem (2018) 293:15070–83. 10.1016/S0304-3940(02)01423-4 PMC6166732 30111591

[82] Yoo Y-G Yeo MG Kim DK Park H Lee M-O Novel function of orphan nuclear receptor Nur77 in stabilizing hypoxia-inducible factor-1alpha. J Biol Chem (2004) 279:53365–73. 10.1074/jbc.M408554200 15385570

[83] Forristal CE Nowlan B Jacobsen RN Barbier V Walkinshaw G Walkley CR HIF-1α is required for hematopoietic stem cell mobilization and 4-prolyl hydroxylase inhibitors enhance mobilization by stabilizing HIF-1α. Leukemia (2015) 29:1366–78. 10.1038/leu.2015.8 PMC4498452 25578474

[84] Williams MA Ravkov EV Bevan MJ Rapid Culling of the CD4+ T Cell Repertoire in the Transition from Effector to Memory. Immunity (2008) 28:533–45. 10.1016/j.immuni.2008.02.014 PMC2391296 18356084

[85] Leignadier J Labrecque N Epitope density influences CD8+ memory T cell differentiation. PloS One (2010) 5:e13740. 10.1371/journal.pone.0013740.g001 21060788 PMC2966420

[86] Richard AC Lun ATL Lau WWY Göttgens B Marioni JC Griffiths GM T cell cytolytic capacity is independent of initial stimulation strength. Nat Immunol (2018) 19:849–58. 10.1038/s41590-018-0160-9 PMC6300116 30013148

[87] Bending D Martín PP Paduraru A Ducker C Marzaganov E Laviron M A timer for analyzing temporally dynamic changes in transcription during differentiation in vivo. J Cell Biol (2018) 217:jcb.201711048. 10.1002/eji.201141702 PMC6080944 29941474

[88] Liu X Wang Y Lu H Li J Yan X Xiao M Genome-wide analysis identifies NR4A1 as a key mediator of T cell dysfunction. Nature (2019) 567:525–9. 10.1038/s41586-019-0979-8 PMC6507425 30814730

[89] Odagiu L Boulet S Maurice De Sousa D Daudelin J-F Nicolas S Labrecque N Early programming of CD8+ T cell response by the orphan nuclear receptor NR4A3. Proc Natl Acad Sci USA (2020) 117:24392–402. 10.1073/pnas.2007224117 PMC7533658 32913051

[90] Ashouri JF Weiss A Endogenous Nur77 Is a Specific Indicator of Antigen Receptor Signaling in Human T and B Cells. J Immunol (2017) 198:657–68. 10.4049/jimmunol.1601301 PMC5224971 27940659

[91] Best JA Blair DA Knell J Yang E Mayya V Doedens A Transcriptional insights into the CD8(+) T cell response to infection and memory T cell formation. Nat Immunol (2013) 14:404–12. 10.1038/ni.2536 PMC3689652 23396170

[92] Mackay LK Rahimpour A Ma JZ Collins N Stock AT Hafon M-L The developmental pathway for CD103(+)CD8+ tissue-resident memory T cells of skin. Nat Immunol (2013) 14:1294–301. 10.1038/ni.2744 24162776

[93] Beura LK Wijeyesinghe S Thompson EA Macchietto MG Rosato PC Pierson MJ T Cells in Nonlymphoid Tissues Give Rise to Lymph-Node-Resident Memory T Cells. Immunity (2018) 48:327–338.e5. 10.1016/j.immuni.2018.01.015 29466758 PMC5828517

[94] Kurd NS He Z Louis TL Milner JJ Omilusik KD Jin W Early precursors and molecular determinants of tissue-resident memory CD8+ T lymphocytes revealed by single-cell RNA sequencing. Sci Immunol (2020) 5 eaaz6894 10.1126/sciimmunol.aaz6894 32414833 PMC7341730

[95] Milner JJ Toma C Yu B Zhang K Omilusik K Phan AT Runx3 programs CD8+ T cell residency in non-lymphoid tissues and tumours. Nature (2017) 552:253–7. 10.1038/nature24993 PMC5747964 29211713

[96] Martinez GJ Pereira RM Äijö T Kim EY Marangoni F Pipkin ME The transcription factor NFAT promotes exhaustion of activated CD8^+^ T cells. Immunity (2015) 42:265–78. 10.1016/j.immuni.2015.01.006 PMC4346317 25680272

[97] Giordano M Henin C Maurizio J Imbratta C Bourdely P Buferne M Molecular profiling of CD8 T cells in autochthonous melanoma identifies Maf as driver of exhaustion. EMBO J (2015) 34:2042–58. 10.15252/embj.201490786 PMC4551351 26139534

[98] Yang R Cheng S Luo N Gao R Yu K Kang B Distinct epigenetic features of tumor-reactive CD8+ T cells in colorectal cancer patients revealed by genome-wide DNA methylation analysis. Genome Biol (2019) 21:2. 10.1186/s13059-019-1921-y 31892342 PMC6937914

[99] Seo H Chen J González-Avalos E Samaniego-Castruita D Das A Wang YH TOX and TOX2 transcription factors cooperate with NR4A transcription factors to impose CD8+ T cell exhaustion. Proc Natl Acad Sci USA (2019) 116:12410–5. 10.1073/pnas.1905675116 PMC6589758 31152140

[100] Boddupalli CS Nair S Gray SM Nowyhed HN Verma R Gibson JA ABC transporters and NR4A1 identify a quiescent subset of tissue-resident memory T cells. J Clin Invest (2016) 126:3905–16. 10.1172/JCI85329DS1 PMC5096804 27617863

[101] Wherry EJ Kurachi M Molecular and cellular insights into T cell exhaustion. Nat Rev Immunol (2015) 15:486–99. 10.1038/nri3862 PMC4889009 26205583

[102] Utzschneider DT Charmoy M Chennupati V Pousse L Ferreira DP Calderon-Copete S T Cell Factor 1-Expressing Memory-like CD8. Immunity (2016) 45:415–27. 10.1016/j.immuni.2016.07.021 27533016

[103] Miller BC Sen DR Abosy Al R Bi K Virkud YV LaFleur MW Subsets of exhausted CD8+ T cells differentially mediate tumor control and respond to checkpoint blockade. Nat Immunol (2019) 20:326–36. 10.1038/s41590-019-0312-6 PMC6673650 30778252

[104] Im SJ Hashimoto M Gerner MY Lee J Kissick HT Burger MC Defining CD8+ T cells that provide the proliferative burst after PD-1 therapy. Nature (2016) 537:417–21. 10.1038/nature19330 PMC5297183 27501248

[105] Wang D Diao H Getzler AJ Rogal W Frederick MA Milner J The Transcription Factor Runx3 Establishes Chromatin Accessibility of cis-Regulatory Landscapes that Drive Memory Cytotoxic T Lymphocyte Formation. Immunity (2018) 48:659–674.e6. 10.1016/j.immuni.2018.03.028 29669249 PMC6750808

[106] Saijo K Winner B Carson CT Collier JG Boyer L Rosenfeld MG A Nurr1/CoREST pathway in microglia and astrocytes protects dopaminergic neurons from inflammation-induced death. Cell (2009) 137:47–59. 10.1016/j.cell.2009.01.038 19345186 PMC2754279

[107] Yao S Buzo BF Pham D Jiang L Taparowsky EJ Kaplan MH Interferon regulatory factor 4 sustains CD8(+) T cell expansion and effector differentiation. Immunity (2013) 39:833–45. 10.1016/j.immuni.2013.10.007 PMC3855863 24211184

[108] Grusdat M McIlwain DR Xu HC Pozdeev VI Knievel J Crome SQ IRF4 and BATF are critical for CD8^+^ T-cell function following infection with LCMV. Cell Death Differ (2014) 21:1050–60. 10.1038/cdd.2014.19 PMC4207473 24531538

[109] Man K Miasari M Shi W Xin A Henstridge DC Preston S The transcription factor IRF4 is essential for TCR affinity–mediated metabolic programming and clonal expansion of T cells. Nat Immunol (2013) 14:1155–65. 10.1038/ni.2710 24056747

[110] Raczkowski F Ritter J Heesch K Schumacher V Guralnik A Höcker L The transcription factor Interferon Regulatory Factor 4 is required for the generation of protective effector CD8+ T cells. Proc Natl Acad Sci USA (2013) 110:15019–24. 10.1073/pnas.1309378110 PMC3773801 23980171

[111] Nayar R Schutten E Bautista B Daniels K Prince AL Enos M Graded Levels of IRF4 Regulate CD8+ T Cell Differentiation and Expansion, but Not Attrition, in Response to Acute Virus Infection. J Immunol (2014) 192 (12):5881–93 10.4049/jimmunol.1303187 PMC4080788 24835398

[112] Ma W Zhao R Yang R Liu B Chen X Wu L Nuclear receptors of the NR4a family are not required for the development and function of follicular T helper cells. Int Immunopharmacol (2015) 28:841–5. 10.1016/j.intimp.2015.04.012 25899083

[113] Sekiya T Kondo T Shichita T Morita R Ichinose H Yoshimura A Suppression of Th2 and Tfh immune reactions by Nr4a receptors in mature T reg cells. J Exp Med (2015) 144:1623–40. 10.1016/S1074-7613(03)00292-9 PMC4577835 26304965

[114] Doi Y Oki S Ozawa T Hohjoh H Miyake S Yamamura T Orphan nuclear receptor NR4A2 expressed in T cells from multiple sclerosis mediates production of inflammatory cytokines. Proc Natl Acad Sci USA (2008) 105:8381–6. 10.1073/pnas.0803454105 PMC2426110 18550828

[115] Raveney BJE Oki S Yamamura T Nuclear Receptor NR4A2 Orchestrates Th17 Cell-Mediated Autoimmune Inflammation via IL-21 Signalling. PloS One (2013) 8:e56595. 10.1371/journal.pone.0056595.s004 23437182 PMC3578929

[116] Satoh J-I Nakanishi M Koike F Onoue H Aranami T Yamamoto T T cell gene expression profiling identifies distinct subgroups of Japanese multiple sclerosis patients. J Neuroimmunol (2006) 174:108–18. 10.1016/j.jneuroim.2006.02.004 16564577

[117] Harant H Lindley IJD Negative cross-talk between the human orphan nuclear receptor Nur77/NAK-1/TR3 and nuclear factor-kappaB. Nucleic Acids Res (2004) 32:5280–90. 10.1093/nar/gkh856 PMC521667 15466594

[118] Takeuchi Y Nishikawa H Roles of regulatory T cells in cancer immunity. Int Immunol (2016) 28:401–9. 10.1093/intimm/dxw025 PMC4986235 27160722

[119] Facciabene A Motz GT Coukos G T-regulatory cells: key players in tumor immune escape and angiogenesis. Cancer Res (2012) 72:2162–71. 10.1158/0008-5472.CAN-11-3687 PMC3342842 22549946

[120] Sato E Olson SH Ahn J Bundy B Nishikawa H Qian F Intraepithelial CD8+ tumor-infiltrating lymphocytes and a high CD8+/regulatory T cell ratio are associated with favorable prognosis in ovarian cancer. Proc Natl Acad Sci USA (2005) 102:18538–43. 10.1073/pnas.0509182102 PMC1311741 16344461

[121] Hibino S Chikuma S Kondo T Ito M Nakatsukasa H Omata-Mise S Inhibition of Nr4a Receptors Enhances Antitumor Immunity by Breaking Treg-Mediated Immune Tolerance. Cancer Res (2018) 78:3027–40. 10.1158/0008-5472.CAN-17-3102 29559474

